# Development of a Flavor Ingredient Wheel Linking E-Liquid Additives to the Labeled Flavor of Vaping Products

**DOI:** 10.3390/toxics12050372

**Published:** 2024-05-18

**Authors:** Kelly Buettner-Schmidt, Katherine Steward, Maciej L. Goniewicz, Kolby Schaeffer Fraase, Megan Orr, Donald R. Miller

**Affiliations:** 1 School of Nursing, North Dakota State University, Fargo, ND 58108, USA; kolby.schaeffer.1@ndsu.edu (K.S.F.); megan.orr@ndsu.edu (M.O.); donald.r.miller@ndsu.edu (D.R.M.); 2 Department of Chemistry and Biochemistry, Montana State University, Bozeman, MT 59717, USA; kfsteward@gmail.com; 3 Department of Health Behavior, Roswell Park Comprehensive Cancer Center, Buffalo, NY 14263, USA; maciej.goniewicz@roswellpark.org

**Keywords:** e-cigarettes, electronic cigarettes, electronic nicotine delivery systems, vaping, e-liquids, flavoring, nicotine, public health, ingredients, chemicals

## Abstract

E-liquids contain combinations of chemicals, with many enhancing the sensory attractiveness of the product. Studies are needed to understand and characterize e-liquid ingredients, particularly flavorings, to inform future research and regulations of these products. We identified common flavor ingredients in a convenience sample of commercial e-liquids using gas chromatography–mass spectrometry. E-liquid flavors were categorized by flavor descriptors provided on the product packaging. A Flavor Ingredient Wheel was developed to link e-liquid flavor ingredients with flavor categories. An analysis of 109 samples identified 48 flavor ingredients. Consistency between the labeled flavor and ingredients used to produce such flavor was found. Our novel Flavor Ingredient Wheel organizes e-liquids by flavor and ingredients, enabling efficient analysis of the link between ingredients and their flavor profiles and allowing for quick assessment of an e-liquid ingredient’s flavor profile. Investigating ingredient profiles and identifying and classifying commonly used chemicals in e-liquids may assist with future studies and improve the ability to regulate these products.

## 1. Introduction

Many flavoring chemicals are added to e-liquids to increase attractiveness in taste and smell. Although oral ingestion of these compounds may be generally recognized as safe (GRAS) [1], inhalation safety is unknown because GRAS designation does not include an assessment of inhalation risks [2]. Inhalation of complex mixtures, like flavored e-cigarette aerosols, may potentially cause adverse health effects, including respiratory tract inflammation through a variety of mechanisms [1,3,4]. Observed respiratory effects associated with flavored e-liquids may be less than with tobacco smoke but may depend on the specific flavoring chemicals used [1,3,4].

Numerous chemicals have been reported in e-cigarette products [1]. Many previous studies have quantified the number of chemicals, although they all suffered limitations on the sampling or analytical methods [5]. Hutzler et al. [6] found 141 flavor chemicals in 28 e-liquids, and Girvalki et al. [7] found 171 chemicals in 122 samples; however, others have criticized these works, as Hutzler [6] included some samples with ethylene glycol as a solvent and Girvalki [7] only used a quantitative analysis [8]. A review [9] found 9 studies investigating 670 flavored e-liquids and detected between 1 and 47 chemicals per sample, with the most common being ethyl maltol, ethyl vanillin, vanillin, cinnamaldehyde, and menthol. Most recently, Omaiye et. al. found 126 flavor chemicals in 103 bottles of refill liquid manufactured by one company [10]. 

Thus, it is important to have a better understanding of e-liquid chemicals. This study aimed to analyze e-liquid samples purchased within North Dakota, and to identify flavor chemicals used in the various retail flavored e-liquids to inform future research and potential regulations of these products. This work was an exploratory effort to determine as many ingredients as possible in a wide range of products without prior knowledge of these compounds. We then mapped the relationship between flavor descriptors and flavor chemicals to create a new classification system for ingredients, which we call the Flavor Ingredient Wheel.

## 2. Materials and Methods

### 2.1. Sampling of E-Liquids for Analysis 

As part of a more extensive study, e-liquids (*n* = 285; free-base, salts, and nicotine-free) were purchased in 2019 from 35 licensed and unlicensed tobacco specialty stores (vape shops, 45.7%; head shops [shops selling paraphernalia related to recreational drug use, music, countercultural art, etc.] [11], 28.6%; tobacco shops, 17.1%; and other shops, 8.6%) across North Dakota, USA. Shop sampling is described elsewhere [12]. Excluded were retail shops located on American Indian reservations, convenience stores, gas stations, grocery stores, and other similar venues. 

Data collectors purchased common strengths of free-base e-liquids (0, 6, 12, 18, 24, and 36 mg/mL). Less common concentrations (3 or 9 mg/mL), uniquely packaged e-liquids, and e-liquids compounded in-shop were purchased when possible. Data collectors purchased different brands for each concentration, with six e-liquids per shop minimum. Data collectors purchased nicotine salts in three varying concentrations. Samples were stored in the original packaging in a resealable bag in a dark space at room temperature. 

Due to financial constraints, we needed to limit the number of samples for chemical analysis. Of the 285 total e-liquids purchased, 75 e-liquids were randomly sampled. Additionally, some of the 285 samples that were compounded in-shop (*n* = 34) or uniquely packaged (*n* = 14) that were not already in the random sample were also included (*n* = 24 and *n* = 10, respectively). Uniquely packaged samples were defined as those not in containers resembling small bottles, with outer packaging resembling everyday items, or were not standard rectangular packages, resulting in 109 samples being analyzed. 

### 2.2. Overall Sample Analysis Process

**Figure 1:**
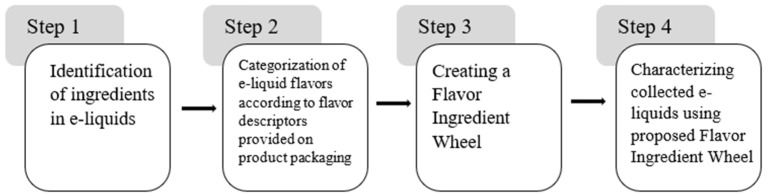
Overall schematic of the study.

Figure 1 provides a schematic for the e-liquid and flavor analysis and the development of our Flavor Ingredient Wheel. 

#### 2.2.1. Step 1: Identification of E-Liquid Flavor Ingredients

Flavoring chemicals and other organic substances were quantitatively measured within three months after purchase. The derivatization method, based on published methods [13,14], was used as follows: methanol (500 μL) was added to 100 μL of 1:100 methanol–diluted e-liquid samples, and the samples were vortexed vigorously for 20 s. N-methyl-N-tert-butyldimethylsilyl-trifluoroacetamide (MTBSTFA; 500 μL) was added to the samples to help ionization of the different molecules, which were vortex mixed for 15 s. Samples were then heated at 80 °C for 3 h. Samples were injected on the Agilent 7890 Gas Chromatography–Mass Spectrometry (GC-MS) system using the 30-m, 0.25-μm film thickness Agilent HP-5ms column (Agilent, product #19091S-433, Santa Clara, CA, USA). Two µL of the material were injected using a 10:1 split method, with instrument conditions as follows: inlet held at 325 °C, with a gas flow rate of 1.5 mL/min. The oven temperature profile was 35 °C for 0 min, then a ramp of 20 °C/min to 320 °C, with a hold for 1 min. The transfer line to the MS was held at 325 °C. The MS scan method was from 35 to 450 *m*/*z*. Samples were analyzed using the Agilent MassHunter Quantitative Analysis software tool (Version 4). Data collection from MS started after three minutes post-injection as the peak of the propylene glycol with short retention time interfered with the collection of the other smaller peaks. Peaks were identified using the 2008 National Institute of Standards and Technology (NIST) [15] standard library spectrum matching. The following was used for matching criteria: molecules had to match at a probability score of 50% using the NIST 08 software, which utilized major ion fragment patterns for comparison [16,17]. Due to the number of mass features present in the samples compared to the reference spectra, this wider tolerance was utilized. Major and unique ions were utilized from butterfly plots to assess match quality. Data were manually curated for NIST matches and identifications that were less than 50% were excluded from the analysis. Ingredients were identified using NIST information and orthogonal data, including retention time, ion fragmentation patterns, and exact mass matching, and were quantified using peak area integration. To determine the limit of detection (LOD) and limit of quantitation (LOQ), US Food and Drug Administration (FDA) and Environmental Protection Agency guidelines and recommendations were used [18,19]. Because this was an untargeted approach, no standards with calibration curves were injected, and the samples were curated manually for LOD. The peaks with the lowest abundance but still 10× the baseline (manual assessment, chromatograms available upon request) for a given analyte were used as the basis for the 3× LOD calculation to reach LOQ. These 3× LOD values are listed in Supplemental Table S1. 

To expand the approach to this analysis, global trends were also evaluated. To achieve this, the relative abundance for each flavoring chemical was compared between samples. This approach assessed trends in the samples by characterizing the ingredients that were present but not seen at our determined LOQ. We wanted to capture all ingredients in each sample, even low concentrations because we acknowledge that the analysis techniques utilized here likely needed to include low-abundant ingredients. This work was intended as an exploratory effort to determine as many ingredients as possible without prior knowledge of these compounds. The MTBSTFA derivatization method selects for active hydrogen molecules and thus yields results for the more polar molecules in the samples. This precluded utilizing standards for absolute quantitation and exact calculations for LOD or LOQ, undoubtedly resulting in ingredients not captured in this analysis.

Chemical ingredients were first identified using the NIST database [15]; the naming schema of NIST was used for original annotation. Chemical names, chemical functional groups, and properties of each ingredient were annotated using databases such as PubChem [20], the Human Metabolome Database [21], the Good Scents Company Information System (Good Scents) [22], and the Flavor Extract Manufacturers Association Flavor Ingredient Library (FEMA) [23]. Functional group classifications were based on super class information from these databases, mostly HMDB. For example, Vanillin acetate’s super class chemical taxonomy is listed as benzenoid under the kingdom of organic compounds, so it is listed as a benzenoid and a cyclic molecule in our table [24]. Ingredients were categorized and defined using the International Union of Pure and Applied Chemistry (IUPAC) [25] name, common name, Chemical Abstracts Services (CAS) [26] number, FEMA [23] number, HMDB number [21] and flavor profile details as described from these sources. IUPAC standardizes nomenclature and terminology for presenting scientific results [25].

#### 2.2.2. Step 2: Categorization of E-Liquid Flavors According to Flavor Descriptors Provided on Product Packaging

E-liquids were categorized as purchased from vape shops, head shops, tobacco shops, and “other” shops. E-liquids were categorized as free-base nicotine salts or nicotine-free based on labels or information provided during purchase. E-liquids were also categorized as compounded or not, either made entirely on-site or when the shop staff added nicotine [12].

The labeled flavor categories were used to report class frequencies, percentages, and groups for the statistical analysis regarding the number of ingredients and relative abundance of identified chemicals and ingredients. Labeled flavor categories included alcohol, candy, coffee/tea, dessert, fruit, menthol/mint, and nuts. Additional categories included other beverages, other flavors, and other sweets. Spices, tobacco, and unflavored were assessed; no samples had these categories. Labels without specific ingredients listed were classified as “unknown flavors”. E-liquid sample labels were also evaluated for references about “cooling” or menthol in their name and/or description or no cooling claim. Cooling claim terms included menthol, mint, ice, freeze, blast, mist, cooler, frost, frozen drink (e.g., daiquiri), mojito, popsicle, slushie (drink), and blue razz.

#### 2.2.3. Step 3: Development of the Flavor Ingredient Wheel

We developed a novel Flavor Ingredient Wheel to organize the reporting of chemical ingredients and the flavor categories of the products. The Flavor Ingredient Wheel provides a visual interpretation of the chemical analysis (Step 1) and flavor product descriptors (Step 2). The outer layer of the wheel includes common flavor ingredients identified in analyzed products (see Step 1 above). The middle layer provides ingredient-based flavor descriptors provided in FEMA [23] and Good Scents [22] databases. The inner (central) layer of the wheel includes six primary flavor categories, as identified in Step 2. The primary flavor categories included in the inner layer of newly developed the Flavor Ingredient Wheel were compared with the previously published Flavor Wheel [27]. 

#### 2.2.4. Step 4: Characterizing E-Liquids Using the Proposed Flavor Ingredient Wheel

We conducted two functional tests of the Flavor Ingredient Wheel. First, we confirmed the label flavor description with the chemical ingredient properties using a random sample of five collected e-liquids. Second, we chose two e-liquid samples without an identifiable flavor profile based on their labels and estimated a flavor profile. 

For a confirmation test, we assessed whether label flavor descriptions were reflected by the flavor ingredients in five samples. The chemical composition of each sample was evaluated, and a potential e-liquid flavor was identified using our Flavor Ingredient Wheel. Each flavor ingredient was placed on our Flavor Ingredient Wheel’s outer layer, which relates to the additional flavor descriptors found in the second layer of the Flavor Ingredient Wheel and were compared with the e-liquid product labels. From there, a primary categorization of an overall flavor is noted by referencing the inner layer of the Flavor Ingredient Wheel. 

We tested whether flavor categories of two unknown flavor samples could potentially be identified based on detected flavor ingredients. For this test, we chose two products with unknown flavors, with names such as “Original Bold”, that did not have a discernible flavor profile on the label. 

### 2.3. Statistical Analysis

To evaluate how well the 109 e-liquid samples used in this study represent the larger pool of 285 e-liquid samples from which they were drawn, chi-square tests of association and Fisher’s exact tests were implemented to compare the distributions of each sample categorization between the e-liquid samples used in this study and the remaining 176 e-liquid samples. An initial analysis was performed on the number of chemical ingredients detected. Then, using the relative abundances (of any amount) for each ingredient, further comparative statistical analysis was performed to parse the more subtle trends and changes in the data due to the categorical factors of interest. For the study of the number of ingredients, one- and two-way analysis of variance (ANOVA) was performed to determine significant differences in group mean among groups in various sample categorizations. The global analysis of abundance data was performed using principal component analysis (PCA), heatmaps with hierarchical clustering, partial least squares discriminant analysis (PLS-DA), and one-way ANOVA. Group comparisons of the sample categorizations previously mentioned were performed in the global study. Statistical significance was set at *p* < 0.05. Statistical analysis was performed in R Version 3.6.2 (using the base and MetaboAnalyst [28] packages) and Excel 2016.

## 3. Results

### 3.1. Sample Characterization

**Figure 2:**
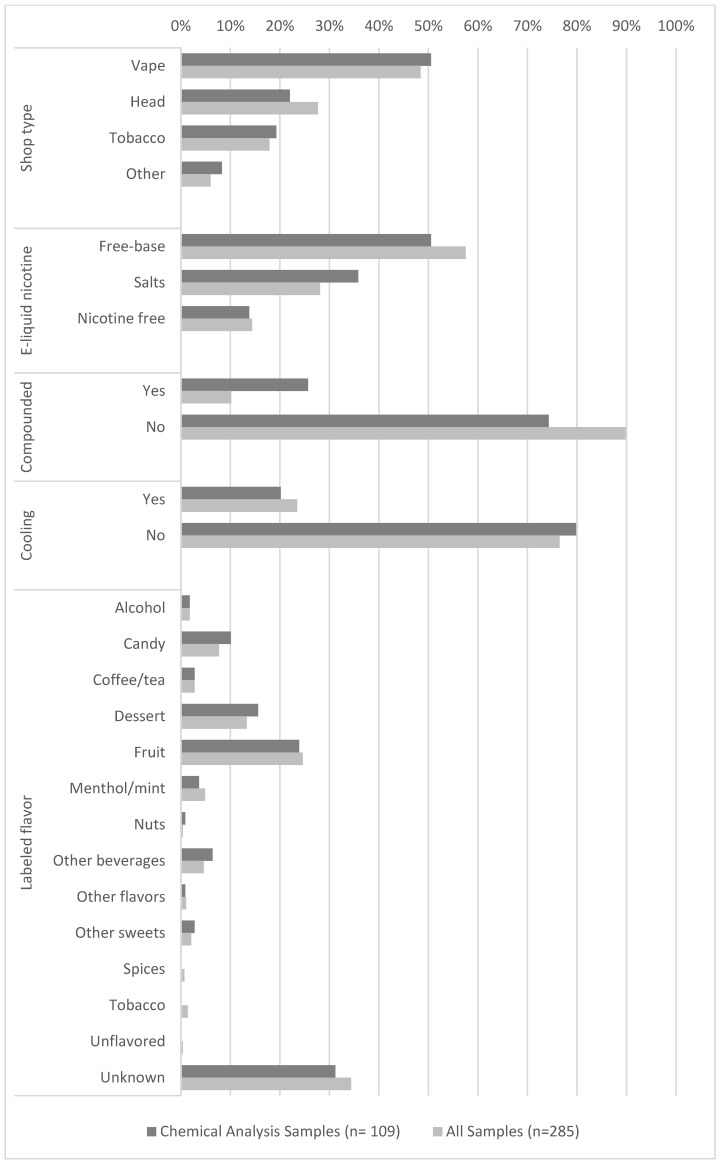
Characterization of e-liquids products analyzed for flavor ingredients.

Figure 2 (also see Table A1) provides the complete sample characteristics of the 109 e-liquid samples analyzed for flavor ingredients in this study and the pool of all 285 e-liquid samples from which the study samples were randomly selected. Except for the compounded categorization, no significant differences were found in the distributions of each sample categorization between the e-liquid samples used in this study and the remaining 176 e-liquid samples from the larger pool of samples. The distributions of the two groups differed significantly for the compounded categorization (*p* < 0.001) due to all compounded samples from the original collection being selected for ingredient analysis as described above. 

The majority of e-liquids were categorized as purchased from vape shops (50.5%), followed by head shops (22.0%), tobacco shops (19.3%), and “other” shops (8.3%). The majority of e-liquids were categorized as free-base (50.5%), followed by nicotine salts (35.8%) and nicotine-free (13.8%) based upon labels or information provided during purchase. About one-quarter (25.7%) of e-liquids were categorized as compounded. Approximately one-fifth (20.2%) of e-liquid sample labels referenced “cooling” or menthol in their name and/or description. 

In terms of the labeled flavor categories, excluding e-liquids without specific ingredients provided on the label (31.2%), fruit was the most common flavor category (23.9%), followed by dessert (15.6%), and candy (10.1%). The less commonly occurring flavor categories included other beverages (6.4%), menthol/mint (3.7%), coffee/tea (2.8%), other sweets (2.8%), alcohol (1.8%), nuts (0.9%), and other flavors (0.9%). No e-liquid samples had labeled flavor categories of spices, tobacco, or unflavored.

### 3.2. Step 1: Common E-Liquid Flavor Chemicals Identified in Analyzed E-Liquids

After analysis using GC-MS and NIST [15] compound matching, 48 ingredients were identified from the 109 samples. Table 1 lists their IUPAC chemical names [25], common names, HMDB numbers [21], chemical super classes (as annotated by databases), chemical functional groups (as annotated by databases), flavor profiles (as annotated by databases), CAS numbers [26], FEMA numbers [23], and substance descriptions (as annotated by databases). Two ingredients were categorized as derivatives of a parent compound because they had the same FEMA number [23], although they had different CAS numbers [26]. These ingredients are denoted on the table and were not annotated in detail as the flavor profile was not unique to the other identified ingredients. A supplementary Excel document (Revised Table 1 with Full Reference Hyperlinks) also denotes this information and includes hyperlinks to each individual ingredient reference.

**Figure 3:**
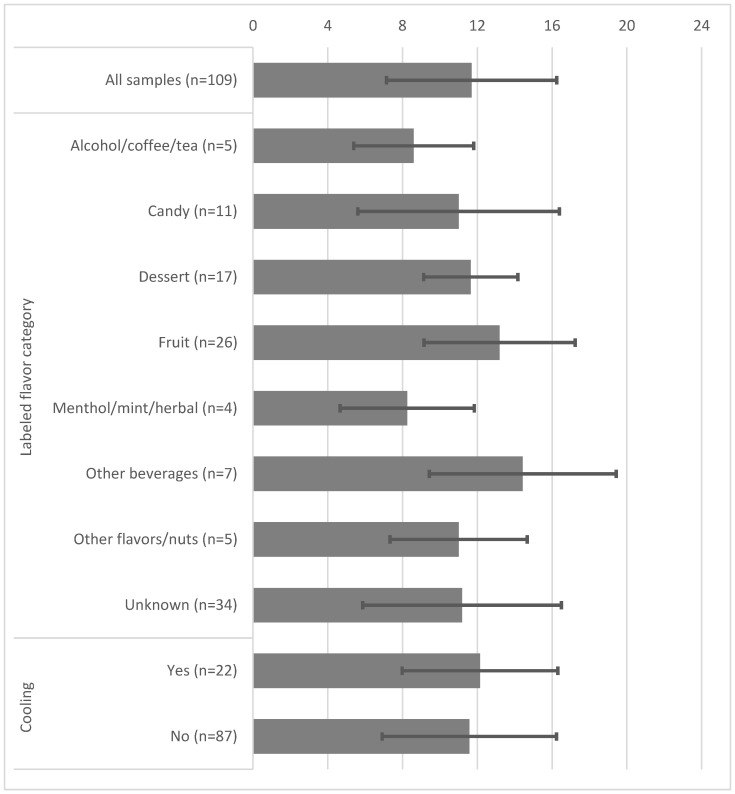
The mean number of ingredients with standard deviation error bars by e-liquid categories.

Of all samples tested, the mean number of flavor ingredients was 11.7 (standard deviation = 4.6). The mean number of ingredients in salt-based e-liquids (15.2 ± 3.4) was significantly greater than either free-base (9.8 ± 4.1; *p* < 0.001) or nicotine-free samples (9.5 ± 3.4; *p* < 0.001) but did not differ significantly among the groups for any other categorical factors (Figure 3 and Table A2).

To ascertain the average chemical makeup of each flavor group based on GC-MS quantitative analysis, the mean number of flavor ingredients, categorized by the identified functional group, was summed, and the distribution was plotted (Supplemental Figure S1) relative to the number of samples in the flavor group, allowing visualization of the most common chemicals per flavor. The fruit-based flavor and unknown-flavor profiles had similar ratios of chemical groups. The candy and alcohol/coffee/tea flavor groups had a similar ratio of aldehydes to terpenoid ingredients. The menthol/mint/herbal and other beverage groups had the most terpenoid ingredients relative to other ingredient classes. The single nut flavor sample, combined with different flavors because there was just one sample in the nut category, was the only sample without an acid-classified ingredient.

The ANOVA showed 15 ingredients with significantly different mean relative abundances among the flavor groups (Supplemental Figure S2 and Supplemental Table S2). The PCA and PLS-DA did not show clear differentiation among the flavor groups, but the heatmap of all the samples and ingredients (Supplemental Figure S3) showed some trends when using only the top 15 ingredients that ANOVA differentiated. When considering only these ingredients, a higher abundance of vanilla (benzenoid and aldehyde compounds) and nut-flavored (alcohol and aldehydes) ingredients in the dessert and coffee/tea category were observed, and herbal and terpenoid ingredients were more abundant in the other beverage and menthol/mint/herbal categories.

The label claim of a cooling sensation revealed patterns among the samples. For the global abundance comparison, ANOVA showed nine ingredients, with significant differences between samples that claimed to cool and samples that did not. The ingredient with the most significant difference in mean relative abundance was cyclohexanol, 1-methyl-4-(1-methylethyl), or menthol, which is consistent with the claims because the cooling sensation comes from menthol. Following menthol, hexanoic acid, butyl ester (glycol, with a fruit flavor profile), and vanillin acetate were the following most significantly differentiated ingredients between the cooling sample types (Supplemental Table S3), with mean relative abundance lower in e-liquids with a cooling claim. The PLS-DA showed separation in component distributions between the cooling and non-cooling samples, although there was some overlap (Supplemental Figure S4). As with the ANOVA, the VIP scores showed that menthol was the ingredient that most discriminated the groups, followed by vanillin, acetate (benzenoid), and 4H-pyran-4-one, 2-ethyl-3-hydroxy (ethyl maltol) (aldehyde). In summary, samples that had cooling ingredients had fewer fruit or floral flavor ingredients and more additives known to provide a cooling sensation, including cyclohexanol, 1-methyl-4-(1-methylethyl) (menthol), and less of other dessert flavors, such as vanillin acetate and the fruity flavor 4H-pyran-4-one, 2-ethyl-3-hydroxy (ethyl maltol).

Supplemental Figure S5 shows the percentage of samples in which other ingredients (non-flavor related) were detected. Nicotine was the most common, seen in 86.2% of the samples, matching the number of samples labeled as containing nicotine. Supplemental Table S4 provides the number of samples with acid type (used to create nicotine salts) and related proportions. Other common ingredients were acetic acid pentyl ester (59.6%), ethyl maltol (58.7%), and benzyl alcohol (56.9%). It should be noted that these data did not characterize propylene glycol, a common solvent used in e-liquids. This ingredient was excluded from the analysis because it overloaded the GC column.

### 3.3. Step 2: Categorization of E-Liquid Flavors Based on Flavor Descriptors Provided on Packaging

Seven flavor categories were identified and annotated from database entries: alcohol/coffee/tea, candy, dessert, fruit, menthol/mint/herbal, other beverages, and other flavors/nuts. We also included an “unknown” flavor category if an ingredient had no flavor reference. “Herbal” was added to menthol/mint based on FEMA [23] and Good Scents [22] information. Some groupings were collapsed. Alcohol was combined with coffee/tea. “Other flavors” and nuts were combined for statistical analyses due to a small sample size.

In terms of the labeled flavor categories, excluding e-liquids without specific ingredients provided on the label (31.2%), fruit was the most common flavor category (23.9%), followed by dessert (15.6%) and candy (10.1%). The less commonly occurring flavor categories included other beverages (6.4%), menthol/mint (3.7%), coffee/tea (2.8%), other sweets (2.8%), alcohol (1.8%), nuts (0.9%), and other flavors (0.9%). No e-liquid samples had labeled flavor categories of spices, tobacco, or unflavored.

### 3.4. Step 3: Flavor Ingredient Wheel

**Figure 4:**
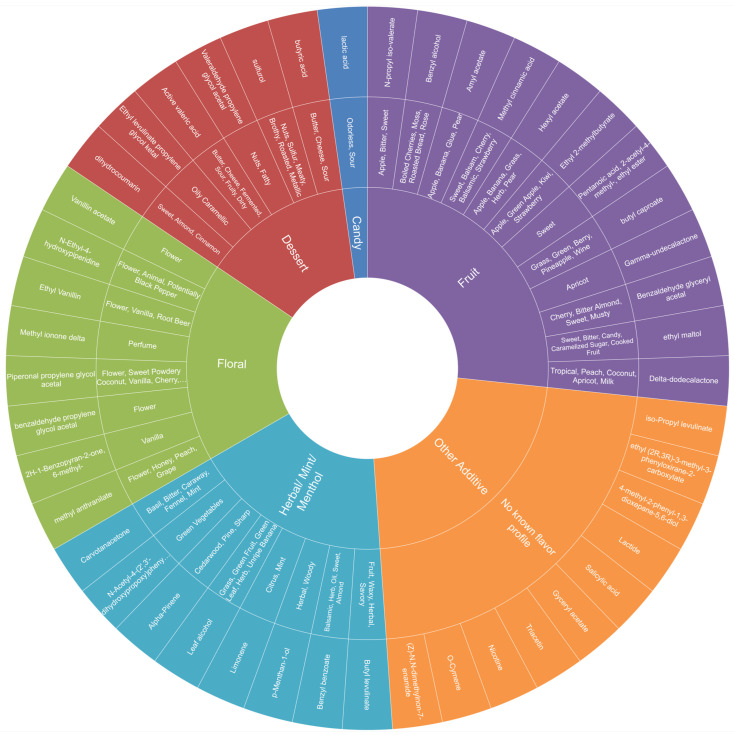
E-Liquid Flavor Ingredient Wheel (Version 1). Note. The e-liquid Flavor Ingredient Wheel categorizes and provides a link between chemistry of the products (flavor ingredients identified in products) and product labelling (flavor category provided on packaging). The outer level includes the common or chemical name of the identified e-liquid ingredients. The middle level identifies the flavor descriptor of each ingredient as described by FEMA Library [23] and Good Scents System database [22]. The inner level categorizes the flavor ingredients similar to Krüsemann’s Flavor Wheel [27].

To illustrate the results, we developed a Flavor Ingredient Wheel (Figure 4), as described in Section 2.2. Our Flavor Ingredient Wheel has three levels. The outer level identified chemicals by common name, or by chemical name if there was no identified common name (see Section 2.2.1). The middle level contains the flavor descriptor as assigned in Section 2.2.1 and Section 2.2.2. The inner level categorizes the flavor ingredients similar to Krüsemann’s flavor categories and includes six primary categories: fruit, candy, dessert, floral, herbal/mint/menthol, and other additives (see Section 2.2.3). The Flavor Ingredient Wheel excludes the following solvents: propylene glycol and glycerin. Table 1 and Table A3 provide the Flavor Ingredient Wheel detailed information.

We compared our Flavor Ingredient Wheel with the Flavor Wheel [27] to assist with the organization of the Flavor Ingredient Wheel’s inner level, and we designed it to be somewhat parallel to the Flavor Wheel [27]. See Supplemental Table S5, Comparison of Primary Flavor Categories.

### 3.5. Step 4: Characterizing E-Liquids Using the Flavor Ingredient Wheel

As shown in Supplemental Table S6, Characterizing Product Flavors Using the Flavor Ingredient Wheel, a dessert sample with a cooling claim contained menthol and other ingredients consistent with a dessert profile, including vanilla flavors and a fruit, herbal, and nutty profile. Without a cooling claim, a second dessert sample had floral, fruit, and nutty ingredients. A fruit-berry sample contained fruit ingredients, and two other fruit-other samples contained primarily fruit ingredients. We tested two samples whose labels provided no flavor profile indication. “Original Bold” had positive detections for acetic acid, pentyl ester (fruit), and ethyl maltol (fruit and cooked sugar), indicating a likely fruity flavor profile. Another unknown flavor, “Zerbert”, could be categorized as a combination of fruit and dessert flavor by evaluating the ingredients of ethyl maltol, ethyl vanillin, and three fruit-flavored ingredients. Using the flavor categories, determined from each ingredient on our Flavor Ingredient Wheel, we could ascertain a rough characterization of these two unknown-flavor samples.

#### Additives Not Associated with Flavors

In addition to the flavor ingredients, we identified major solvents, glyceryl acetate, triacetin, nicotine and organic acids used to create the nicotine salts. The details about acid ingredients are located in the supplementary materials (Supplemental Table S4). Carrier solvents like propylene glycol were excluded from this analysis, as the chromatographic peak was large and interfered with quantitation of other molecules.

## 4. Discussion

The unique Flavor Ingredient Wheel, described for the first time in this paper, helps visualize chemicals in e-liquids and their flavor profiles according to packaging claims. Our Flavor Ingredient Wheel expands on and highlights common flavor ingredients associated with label claims as originally outlined in Krüsemann’s work [27]. Our innovative Flavor Ingredient Wheel is database-sourced and can be applied to methods for categorizing other e-liquids. Investigating ingredient profiles and the frequency of flavor ingredients used in various products has already been demonstrated as a valuable tool when classifying samples and tracking trends in ENDS products. The Flavor Ingredient Wheel allows for a quick assessment of an ingredient’s flavor profile, common name and relationship to package claims. FEMA only provides information about a single compound [23]; thus, it is time-consuming to look up multiple ingredients. The Flavor Ingredient Wheel organizes this information to reference ingredient information quickly but should not replace the detailed information in other databases. We recognize the complexity of identifying flavoring ingredients. Although some ingredients alone may contribute to a single flavor (ex: cherry, menthol), many other flavors are derived from a complex combination of ingredients. A major limitation of the Flavor Ingredient Wheel is that it cannot completely predict a product’s flavor. We strongly recommend that further research be conducted to further explore the complexity of flavoring and revise the Flavor Ingredient Wheel. As the first version of the Flavor Ingredient Wheel, we also recommend that it be updated frequently as ENDS products continue to evolve and as policy impacts the regulation of ingredients and flavors. We encourage other authors to update this figure with their findings.

Overall, we detected 48 different ingredients in 109 e-liquid samples. Two-thirds of our samples had ten or more ingredients. Other studies have shown a range of 1 to 171 ingredients [6,7,10]. In fruit and floral-flavored e-liquids the most identified ingredients were esters and benzenoids, while most dessert-flavored products contained fatty acid derivatives. The most identified ingredients in menthol/mint/herbal were monoterpenes and monoterpenoids.

Vanilla, sweet, and floral ingredients were abundant in the nicotine-free samples. Vanilla and fruit-flavored ingredients were less abundant in e-liquids with a cooling claim. Lactic acid and lactide were more abundant in nicotine salts (Supplemental Table S4).

We used multiple factors to categorize label claims and associated flavor ingredients. Flavor categories were annotated from the detected ingredients based on FEMA [23] and Good Scents [22] descriptions, allowing for a comprehensive evaluation of the e-liquids. Other work, like Scieszka et al. [29], utilized a similar approach to categorize ENDS product small molecules detected in lung tissue using the HMDB and Hsiao et al., 2023 [30] utilized the NIST library to identify unknown molecules and further annotate molecules using the HMDB, a public database that aids other researchers in classifying small molecules [31]. Recent work on user preference based on social media references shows that fruit, sweet, and floral ingredients are users’ most popular flavor profiles [32]. Other evidence indicates that cooling and sweet e-liquids are popular among users, regardless of age [33]. Although our data cannot be extrapolated to all e-liquids, the flavor profile with the most representation was fruit, aligning with these documented e-liquid user preferences [32]. Importantly, we found consistency between labeled flavors and actual ingredients in e-liquids.

Samples that claimed a cooling effect contained menthol, which produces a cooling sensation for the user [33,34]. Mint-flavored tobacco products are preferred where other flavors are difficult to obtain or are prohibited [35]. Calls have been made to remove menthol for over two decades, and a recent proposed rule makes this a reality [36]. Although menthol has not been regulated e-cigarettes yet, menthol is banned in tobacco cigarettes in Canada, the United Kingdom, the European Union, and several other jurisdictions [37,38]. Vanillin acetate and ethyl vanillin were abundant and contributed to the differentiation of samples based on the label’s cooling and flavor claims. Thus, one might hypothesize that the presence of menthol and lack of vanillin suggests that the e-liquid has a “cooling” flavor.

Identifying commonly used flavoring chemicals in e-liquids may assist with future studies and evaluating their inhalation toxicity. All flavor ingredients detected in analyzed products were included on the FDA Generally Recognized as Safe (GRAS) list. It is essential to recognize that the GRAS designation is based on the chemical’s safety profile when ingested. For many ingredients identified in our study, inhalation safety is largely unknown. The FDA has also established a list of harmful and potentially harmful constituents (HPHC) in tobacco products [39,40]. We used the HPHC list to cross-check against our list of identified e-liquid ingredients. The only chemical ingredient our study identified that matched the HPHC list was nicotine. However, we did find several ingredients which are derivatives of chemicals listed on the HPHC list. Those include derivatives of coumarin, benzene, and acetamide. We did not find diacetyl and cinnamaldehyde, two flavoring ingredients previously identified in e-liquids with potential respiratory concerns [10], in any of the products analyzed in our study. The Dutch National Institute for Public Health and the Environment also published an advisory list of substances that should not be added to tobacco products and e-cigarettes [41]. Among the 48 ingredients in our study, there were some chemical classes that overlapped with the advisory list including coumarins, nicotine molecules and sugars.

A study limitation was that all samples were purchased in one state and thus may not include e-liquids available nationwide or globally. The samples were purchased and analyzed in 2019 and therefore may not be representative of those currently on the market. However, it is very likely that the same chemicals are currently being used, as many of the identified ingredients are common chemicals in e-liquid products. Additionally, we used purposeful sampling rather than random sampling. Because this was part of a larger study focused on e-liquids, it did not include pod-style or other ENDS products. All products were analyzed within three months of purchase and unknown/variable manufacturing time. The e-liquid composition may have changed from the manufacturing time (e.g., due to extreme storage conditions), which we could not evaluate in our study. Other significant limitations of our study are that sampling for chemical analysis was not based on label flavors and our analysis of the random sample did not include tobacco flavored products.

Furthermore, because of budgetary constraints, we conducted chemical research using the MTBSTFA derivatization method, which optimized for polar compounds with reactive hydrogen moieties and did not explore other approaches to evaluate unknown chemicals, degradation, and aerosolized products. This approach, while untargeted, does offer bias towards molecules with reactive hydrogens and is a limitation of our analysis. Only chemicals noted in the NIST database were utilized; new compounds not included in the NIST database may have been missed. For instance, synthetic cooling agents like WS-3, WS-23, and Evercool 180 and 190 have been recently reported in e-liquids as substitutes for menthol. Still, the NIST database needs to include their mass spectra data [42,43,44]. In addition, the NIST database does not list hemiacetals that are byproducts of condensation reactions between flavoring chemicals and solvents in e-liquids [45,46,47]. Notably, the LOQ of the method used in our study can vary significantly based on batch run day effects. Here, we utilized an estimation of LOQ, which was manually determined, not based on a neat standard calibration curve, to ensure that molecules in very low abundance would not be classified as present and calibration curve data were not collected, considering this was an exploratory analysis. Despite these limitations, this study provides a necessary step in the future characterization and regulation of ENDS products. Determining these samples’ chemical flavor ingredients, flavor profiles, and trends will help inform future policy on efficient methods for analyzing these products. One avenue for future research might include analyzing the proportion of ingredient integrated areas from chromatograms compared to overall total integrated area of each sample. This is one approach that can speak to the proportion of ingredients within the products [48,49], but was not applicable here, as we excluded the carrier solvent from our analysis and thus a large peak that should be included in such an approach.

The development of straightforward tools such as our Flavor Ingredient Wheel demonstrates that tracking use and trends in ENDS products can be simplified on some levels. Future research recommendations include replication/expansion of this study with more samples, using a random-sampling protocol, include information about the e-liquid brands and their market share, and include additional tobacco products and emerging products to confirm or revise the Flavor Ingredient Wheel.

## 5. Conclusions

In conclusion, this study shows the diversity of flavor ingredients and labeled flavor profiles in e-liquids and suggests a method of organizing them. Trends prevail despite the ambiguity and multiplicity of flavors when analyzing these samples by flavor categories or cooling status and the variety of ingredient profiles. Recent FDA regulations [50] are starting to prohibit the use of fruit- and dessert-flavored e-cigarette liquids. However, the effect of e-liquids on health has yet to be fully understood, and studies such as this one offer tools to better understand the implications of using these products.

## Figures and Tables

**Table 1:** Identification of E-Liquid Chemicals.

Identified Molecule from NIST Library	IUPAC Chemical Name	Common Name	Flavor Profile	Physical Properties	FEMA #	CAS #	HMBD #	Super Class	Chemical Class with Annotation Source
3-Hexen-1-ol, (Z)-	(Z)-hex-3-en-1-ol	leaf alcohol	Grass, green fruit, green leaf, herb, unripe banana	oily liquid	2563	928-96-1 44-12-7	NA	alcohol	fatty alcohol ^1^
1,3-Dioxolane, 4-methyl-2-phenyl-	4-methyl-2-phenyl-1,3-dioxolane	benzaldehyde propylene glycol acetal	Floral	colorless to pale yellow clear oily liquid	2130	2568-25-4	NA	cyclic	benzene and substituted derivatives ^1^
Acetic acid, pentyl ester	pentyl acetate	amyl acetate, isoamyl acetate, isovaleric acid	Apple, banana, glue, pear	clear colorless liquid	2055	628-63-7 123-92-2	39095	ester	carboxylic acid ester ^1^
Butanoic acid, 3-methyl-, ethyl ester ***	NA	isovaleric acid derivative	NA	NA	2055	108-64-5	NA	NA	NA
1-Butanol, 3-methyl-, acetate ***	NA	isoamyl acetate derivative	NA	NA	2055	123-92-2	NA	NA	NA
2-Cyclohexen-1-one, 2-methyl-5-(1-methylethenyl)-, (R)-	(5S)-2-methyl-5-propan-2-ylcyclohex-2-en-1-one	carvotanacetone	Basil, bitter, caraway, fennel, mint	NA	2249	6485-40-1 2244-16-8 99-49-0	35824	cyclic	menthane monoterpenoid ^1^
1,3-Benzodioxole, 5-(4-methyl-1,3-dioxolan-2-yl)-	4-(4-Methyl-1,3-dioxolan2-yl)-1,3-benzodioxole 4-Methyl-2-(3,4-methylenedioxyphenyl)- 1,3-dioxolane	piperonal propylene glycol acetal, or heliotropin	Floral, sweet powdery coconut, vanilla, cherry, spice	pale yellow or colorless liquid	4622	61683-99-6	37286	cyclic	1,3-dioxolanes ^1^
Butyl caproate-	butyl hexanoate	butyl caproate	Fruit, grass, green, berry, pineapple, wine	colorless clear liquid	2201	626-82-4	40211	ester	fatty acid ester ^1^
Butanoic acid, 2-methyl-, ethyl ester	ethyl 2-methylbutanoate	Ethyl 2-methylbutyrate	Apple, ester, green apple, kiwi, strawberry	NA	2443	7452-79-1	33745	ester	fatty acid ester ^1^
Butanoic acid, 2-methyl-	2-methylbutanoic acid	Active Valeric Acid	Butter, cheese, fermented, sour, fruity, dirty	colorless to pale yellow clear liquid	2695	116-53-0		acid	alkyl carboxylic acid ^2^
4H-Pyran-4-one, 2-ethyl-3-hydroxy- Ethyl maltol	2-ethyl-3-hydroxypyran-4-one	ethyl maltol, no. 64	Fruit, sweet, bitter, candy, caramelized sugar, cooked fruit	white solid	3487	4940-11-8	31735	cyclic	pyranone ^1^
Benzene, 1-methyl-2-(1-methylethyl)-	1-methyl-2-propan-2-ylbenzene	O-Cymene	NA	flammable colorless liquid	NA	527-84-4	37050	cyclic	cumene ^1^
2H-1-Benzopyran-2-one, 6-methyl-	6-methyl-4-(morpholin-4-ylmethyl)chromen-2-one	NA	Possibly vanilla	NA	2699	92-48-8	32394	cyclic	coumarin, ketone, benzenoid ^1^
1,3-Dioxolane-2-propanoic acid, 2,4-dimethyl-, ethyl ester	ethyl 3-(2,4-dimethyl-1,3-dioxolan-2-yl)propanoate	Ethyl levulinate propyleneglycol ketal	Oily caramellic	colorless clear liquid	4479	5413-49-0	40433	acid	gamma keto acid ^1^
Veratraldehyde propylene glycol acetal	2-butyl-4-methyl-1,3-dioxolane	Valeraldehyde propyleneglycol acetal	Nuts, fatty	colorless clear liquid	4372	74094-60-3	32551	cyclic	dioxolane ^1^
Ethyl Vanillin	3-ethoxy-4-hydroxybenzaldehyde	Ethyl vanillin, ethyl protal or bourbonal	Floral, vanilla, root beer	white, off white powder	2464	121-32-4	29665	aldehyde	hydroxybenzaldehyde ^1^
Benzyl Alcohol (flavor enhancer)	phenylmethanol	benzyl alcohol	Boiled cherries, moss, roasted bread, rose	clear colorless liquid with a pleasant odor	2137	100-51-6	3119	cyclic	benzyl alcohol, benzenoid ^1^
1,3-Dioxan-5-ol, 2-phenyl- (Benzaldehyde glyceryl acetal derivative)	2-phenyl-1,3-dioxan-5-ol	Benzaldehyde glyceryl acetal benzalglycerin or benzylideneglycerol	Fruit, cherry, bitter almond, sweet, musty	colorless to pale yellow clear oily liquid	2129	1319-88-6 1708-40-3 1708-39-0	32174	cyclic	1,3 dioxane, benzene ^1^
Acetic acid, hexyl ester	hexyl acetate	Hexyl acetate, N-hexyl ethanoate or hexyl acetic acid	Apple, banana, grass, herb, pear	colorless liquid with a mild sweet odor	2565	142-92-7	29980	ester	carboxylic acid ester ^1^
Pentanoic acid, 4-oxo-, 1-methylethyl ester (iso-Propyl levulinate)	propan-2-yl 4-oxopentanoate	iso-Propyl levulinate	NA	colorless clear liquid	NA	21884-26-4	NA	ester	ester ^3^
Butanoic acid (glycerol ester)	butanoic acid	butyric acid	Butter, cheese, sour	oily, colorless liquid with an unpleasant odor	2221	107-92-6	NA	acid	fatty acid ^4^
Benzoic acid, 2-amino-, methyl ester	methyl 2-aminobenzoate	methyl anthranilate	Flower, honey, peach, grape	clear colorless to tan liquid with an odor of grapes	2682	134-20-3	NA	ester	ester ^5^
2-Propenoic acid, 3-phenyl-, methyl ester (Cinnamic acid, methyl ester, (E)-)	methyl 3-phenylprop-2-enoate	Methyl cinnamic acid	Sweet, balsam, cherry, balsamic, strawberry	NA	2698	103-26-4	33833	ester	methyl ester ^1^
Butanoic acid, 3-methyl-, propyl ester	propyl 3-methylbutanoate	N-propyl iso-valerate	Fruit, apple, bitter, sweet	NA	2960	557-00-6	NA	ester	fatty acid ester ^6^
(S)-2-Hydroxypropanoic acid	methoxymethyl (2S)-2-hydroxypropanoate	lactic acid, lactate	Odorless, sour	colorless clear viscous liquid or solid	2611	10326-41-7 79-33-4 50-21-5	1311	acid	alpha hydroxy acids and derivatives, organic acids ^1^
Pentanoic acid, 4-oxo-, butyl ester	butyl 4-oxopentanoate	butyl levulinate	Fruit, waxy, herbal, savory	colorless to pale yellow clear liquid	2207	2052-15-5	40165	acid	gamma keto acid, organic acids and derivatives ^1^
Limonene	1-methyl-4-prop-1-en-2-ylcyclohexene	limonene	Citrus, mint	colorless liquid	1326	5989-27-5	4321	cyclic	monoterpene ^1^
5-Thiazoleethanol, 4-methyl-	2-(4-methyl-1,3-thiazol-5-yl)ethanol	sulfurol	Nuts, sulfur, meaty, brothy, roasted, metallic	colorless to pale yellow clear oily liquid	3204	137-00-8	32985	cyclic	4,5-disubstituted thiazoles, azoles, aromatic, heterocyclic ^1^
Cyclohexanol, 1-methyl-4-(1-methylethyl)-	1-methyl-4-propan-2-ylcyclohexan-1-ol	p-Menthan-1-ol	Herbal, woody	colorless liquid to solid	NA	21129-27-1	37020	cyclic	menthane monoterpenoid, monoterpenoids ^1^
N-Acetyl-4-(2′,3′-dihydroxypropoxy)phenylacetamide	N-acetyl-2-[4-(2,3-dihydroxypropoxy)phenyl]acetamide	NA	Potentially green vegetables	NA	NA	NA	NA	nitrogen	acetamide ^3^
N-Ethyl-4-hydroxypiperidine	1-ethylpiperidin-4-ol	N-Ethyl-4-hydroxypiperidine	Heavy floral, animal, potentially black pepper	colorless fuming liquid	NA	3518-83-0	NA	nitrogen	piperidine, ring structure ^3^
Non-7-enoic acid, dimethylamide	(Z)-N,N-dimethylnon-7-enamide	NA	NA	NA	NA	NIST # 187265	NA	nitrogen	amide ^3^
Triacetin	2,3-diacetyloxypropyl acetate	Triacetin	NA	NA	2007	102-76-1	29592	glycerolipid	TAG, lipid, glycerolipid ^1^
(2(3H)-Furanone, 5-heptyldihydro-)	5-heptyloxolan-2-one	gamma-undecalactone	Apricot, fruit	colorless clear liquid	3091	104-67-6	NA	cyclic	lactone ^5^
Benzyl Benzoate	benzyl benzoate	benzyl benzoate	Balsamic, herb, oil, sweet, and almond	NA	2138	120-51-4	14814	cyclic	benzoic acid ester, benzenoid ^1^
1,2,3-Propanetriol, monoacetate/Acetin	2,3-dihydroxypropyl acetate	glyceryl acetate	NA	colorless clear liquid	NA	100-78-7	31712	glycerolipid	DAG, diester ^3^
1R-a-Pinene	(1R,5R)-2,6,6-trimethylbicyclo[3.1.1]hept-2-ene	alpha-Pinene	Cedarwood, pine, sharp	NA	2902	80-56-8 7785-26-4 7785-70-8	6525	cyclic	terpene ^1^
Salicylic acid	2-hydroxybenzoic acid	salicylic acid	NA	odorless white to light tan solid	3985	69-72-7	NA	cyclic	benzenoid ^7^
2H-1-Benzopyran-2-one, 3,4-dihydro-	6-hydroxy-3,4-dihydrochromen-2-one	dihydrocoumarin, 3,4-dihydrocoumarin or 1,2-benzodihydropyrone	Sweet, almond, and cinnamon	NA	NA	119-84-6	36626	cyclic	coumarin, benzenoid ^3^
Oxiranecarboxylic acid, 3-methyl-3-phenyl-, ethyl ester, cis-	ethyl (2R,3R)-3-methyl-3-phenyloxirane-2-carboxylate	NA	NA	NA	NA	19464-95-0	31729	cyclic	oxirane carboxylic acid, benzene, epoxide ^1^
1-Deoxy-d-arabitol	4-methyl-2-phenyl-1,3-dioxepane-5,6-diol	NA	NA	NA	NA	13942-77-3	41486	alcohol	secondary alcohol ^1^
2H-Pyran-2-one, 6-heptyltetrahydro-	(6R)-6-heptyloxan-2-one	Delta-Dodecalactone	Fruit tropical, peach, coconut, apricot, milk	NA	2401	713-95-1	37116	cyclic	fatty ester lipid, lactone, organoheterocyclic ^1^
Pentanoic acid, 2-acetyl-4-methyl-, ethyl ester	ethyl 2-acetyl-4-methylpentanoate	NA	Sweet and fruity	NA	NA	1522-34-5	31579	ester	fatty acid ester ^1^
Vanillin, acetate	(4-formyl-2-methoxyphenyl) acetate	Vanillin acetate	Floral	NA	3108	881-68-5	29663	cyclic	phenol esters ^1^
3-Buten-2-one, 4-(2,6,6-trimethyl-1-cyclohexen-1-yl)-, (E)-	3-methyl-4-(2,6,6-trimethylcyclohexen-1-yl)but-3-en-2-one	Methyl ionone delta, Beta-Isomethylionine	Floral	NA	4151	79-89-0	6189	cyclic	sesquiterpenoids ^8^
Neonicotine	3-[(2S)-piperidin-2-yl]pyridine	NA	NA	NA	NA	494-52-0	NA	nitrogen	NA
Nicotine-1N′-oxide	3-[(2S)-1-methylpyrrolidin-2-yl]-1-oxidopyridin-1-ium	NA	NA	NA	NA	2820-55-5	NA	nitrogen	NA
Nicotine	3-[(2S)-1-methylpyrrolidin-2-yl]pyridine	NA	NA	NA	NA	54-11-5 22083-74-5	NA	nitrogen	NA

Note. # = number. CAS, Chemical Abstracts Services; FEMA, Flavor Extract Manufacturers Association Flavor Ingredient; IUPAC, International Union of Pure and Applied Chemistry; *** = the first asterisk indicates the “parent” ingredient: the next ones are derivatives of that one; NA = no information provided by a database source. Superscript numbers indicate the Chemical Class annotation source: HDMB = ^1^; IFA = ^2^; PubChem = ^3^; LMBD = ^4^; EBI = ^5^; NP-MRD = ^6^; YMDB = ^7^; ContaminantDB = ^8^. Full citation links for each ingredient and chemical class can be found in the Supplemental Excel Document S1 titled Revised Table 1 with Full Reference Hyperlinks.

## Data Availability

Data files from the mass spectrometry are available at Harvard Dataverse Repository using this link https://doi.org/10.7910/DVN/MWFQD6. An Excel file, Data File Number of Ingred Flavors, is also available in the Supplementary Materials.

## Supplementary Materials

The following supporting information can be downloaded at: https://www.mdpi.com/article/10.3390/toxics12050372/s1, Supplemental Table S1 displays integrated peak area abundances for LOQ estimation. Supplemental Figure S1 provides the average chemical classification breakdown of ingredients by labeled flavor category. Supplemental Figure S2 illustrates ingredients by flavor classification. Supplemental Table S2 lists ingredients with significant mean abundance differences noted among flavor categories. Supplemental Figure S3 is a heatmap showing the relative abundances of ingredients detected in e-liquid samples. Supplemental Table S3 lists ingredients significantly different between groups based on cooling claim. Supplemental Figure S4 provides PLS-DA and VIP scores in the projection of ingredients based on cooling claims on packaging. Supplemental Figure S5 illustrates the distribution of detected e-liquid ingredients. Supplemental Table S4 lists acids identified in e-liquid samples. Supplemental Table S5 provides an identification of e-liquid chemicals. Supplemental Table S6 characterizes product flavors using the Ingredient Wheel. Supplemental Excel Document S1 (Revised Table 1 with Full Reference Hyperlinks) denotes references to all of the databases used to create Table 1 and includes hyperlinks to each individual ingredient reference.

## Appendix A

**Table A1:** Class frequencies and percentages by sample categories.

	Chemical Analysis Samples (*n* = 109)		All Samples (*n* = 285)	
Shop Type	Frequency	Percentage	Frequency	Percentage
Vape	55	50.5	138	48.4
Head	24	22.0	79	27.7
Tobacco	21	19.3	51	17.9
Other	9	8.3	17	6.0
E-liquid nicotine type				
Free-base	55	50.5	164	57.5
Salts	39	35.8	80	28.1
Nicotine free	15	13.8	41	14.4
Compounded				
Yes	28	25.7	29	10.2
No	81	74.3	256	89.8
Cooling				
Yes	22	20.2	67	23.5
No	87	79.8	218	76.5
Labeled flavor category				
Alcohol	2	1.8	5	1.8
Candy	11	10.1	22	7.7
Coffee/tea	3	2.8	8	2.8
Dessert	17	15.6	38	13.3
Fruit	26	23.9	70	24.6
Menthol/mint	4	3.7	14	4.9
Nuts	1	0.9	1	0.4
Other beverages	7	6.4	13	4.6
Other flavors	1	0.9	3	1.1
Other sweets	3	2.8	6	2.1
Spices	0	0.0	2	0.7
Tobacco	0	0.0	4	1.4
Unflavored	0	0.0	1	0.4
Unknown	34	31.2	98	34.4

**Table A2:** Summary statistics for number of ingredients by e-liquid categories.

Characteristic	*N*	Mean	*SD*	Min	Median	Max
All samples	109	11.69	4.55	3	12	22
E-liquid nicotine type						
Free-base	55	9.82	4.09	3	10	19
Salts ^a^	39	15.15	3.41	8	16	22
Nicotine-free	15	9.53	3.38	4	9	15
Compounded						
Yes	28	11.21	4.20	4	12	19
No	81	11.85	4.68	3	102	22
Labeled flavor category						
Alcohol/coffee/tea	5	8.60	3.21	4	9	13
Candy	11	11.00	5.39	4	13	20
Dessert	17	11.65	2.52	8	11	18
Fruit	26	13.19	4.04	5	14.5	20
Menthol/mint/herbal	4	8.25	3.59	5	7.5	13
Other beverages	7	14.43	5.00	8	14	22
Other flavors/nuts	5	11.00	3.67	7	11	16
Unknown	34	11.18	5.31	3	12.5	21
Cooling						
Yes	22	12.14	4.16	5	11.5	19
No	87	11.57	4.66	3	12	22

Note. Max = maximum; min = minimum; *SD* = standard deviation; ^a^ Mean number of ingredients significantly higher for salt samples compared with free-base and nicotine-free samples (*p* < 0.05).

**Table A3:** Chemical functional group ingredients and flavor profile.

Detected Esters	Flavor Profile
Acetic acid, pentyl ester	Apple, banana, glue, pear
Butyl caproate-	Fruit, grass, green, berry, pineapple, wine
Butanoic acid, 2-methyl-, ethyl ester *	Apple, ester, green apple, kiwi, strawberry
Butanoic acid, 3-methyl-, ethyl ester	Apple, ester, green apple, kiwi, strawberry
1-butanol, 3-methyl-, acetate (derivative of *)	Apple, ester, green apple, kiwi, strawberry
1,3-dioxolane-2-propanoic acid, 2,4-dimethyl-, ethyl ester	Oily caramellic
Acetic acid, hexyl ester	Apple, banana, grass, herb, pear
Butanoic acid (glycerol ester)	Butter, cheese, sour
Benzoic acid, 2-amino-, methyl ester	Flower, honey, peach, grape
2-propenoic acid, 3-phenyl-, methyl ester (cinnamic acid, methyl ester, (E)-)	Sweet, balsam, cherry, balsamic, strawberry
Butanoic acid, 3-methyl-, propyl ester	Fruit, apple, bitter, sweet
Oxiranecarboxylic acid, 3-methyl-3-phenyl-, ethyl ester, cis-	None
Pentanoic acid, 2-acetyl-4-methyl-, ethyl ester	Sweet and fruity
Detected aldehydes	Flavor profile
4H-pyran-4-one, 2-ethyl-3-hydroxy-ethyl maltol	Fruit, sweet, bitter, candy, caramelized sugar, cooked fruit
Veratraldehyde propylene glycol acetal	Nuts, fatty
Ethyl vanillin	Floral, vanilla, root beer
1,3-dioxan-5-ol, 2-phenyl- (benzaldehyde glyceryl acetal derivative)	Fruit, cherry, bitter almond, sweet, musty
Detected alcohols	Flavor profile
3-hexen-1-ol, (Z)-	Grass, green fruit, green leaf, herb, unripe banana
5-thiazoleethanol, 4-methyl-	Nuts, sulfur, meaty, broth, roasted, metallic
1-deoxy-d-arabitol	Sweet
Detected glycols, glycerol, or sugars	Flavor profile
1,2,3-propanetriol, monoacetate/acetin	None
1,3-benzodioxole, 5-(4-methyl-1,3-dioxolan-2-yl)-	Floral, sweet powdery coconut, vanilla, cherry, spice
Triacetin	None
Detected acids	Flavor profile
Butanoic acid, 2-methyl-	Butter, cheese, fermented, sour, fruity, dirty
Pentanoic acid, 4-oxo-, 1-methylethyl ester (iso-propyl levulinate)	Oily caramellic
(S)-2-hydroxypropanoic acid	Odorless, sour
1,4-dioxane-2,5-dione, 3,6-dimethyl-	None
Pentanoic acid, 4-oxo-, butyl ester	Fruit, waxy, herbal, savory
Salicylic acid	None
Detected benzenoids or coumarins	Flavor profile
1,3-dioxolane, 4-methyl-2-phenyl-	Floral
Benzene, 1-methyl-2-(1-methylethyl)-	None
Benzyl alcohol (flavor enhancer)	Boiled cherries, moss, roasted bread, rose
Benzyl benzoate	Balsamic, herb, oil, sweet, and almond
Vanillin, acetate	Floral
2H-1-benzopyran-2-one, 6-methyl-	Possibly vanilla
2(3H)-furanone, 5-heptyldihydro-	Apricot, fruit
2H-1-benzopyran-2-one, 3,4-dihydro-	Sweet, almond, and cinnamon
2H-pyran-2-one, 6-heptyltetrahydro-	Fruit tropical, peach, coconut, apricot, milk
Detected terpenoids	Flavor profile
2-cyclohexen-1-one, 2-methyl-5-(1-methylethenyl)-, (R)-	Basil, bitter, caraway, fennel, mint
Limonene	Citrus, mint
Cyclohexanol, 1-methyl-4-(1-methylethyl)-	Herbal, woody
1R-a-pinene	Cedarwood, pine, sharp
3-buten-2-one, 4-(2,6,6-trimethyl-1-cyclohexen-1-yl)-, (E)-	Floral
Detected nitrogen compounds	Flavor profile
N-acetyl-2-[4-(2,3-dihydroxypropoxy)phenyl]acetamide	No documented flavor profile
1-ethylpiperidin-4-ol	No known flavor profile
Piperidine	Heavy floral or animal scent
Z)-N,N-dimethylnon-7-enamide	None
Detected nicotine ingredients	Flavor profile
Nicotine	No documented flavor profile
Nicotine-1N’-oxide	No documented flavor profile

* = the first asterisk indicates the “parent” ingredient: the next ones are derivatives of that one.

## References

[1] National Academies of Sciences, Engineering, and Medicine Public Health Consequences of E-Cigarettes. https://nap.nationalacademies.org/catalog/24952/public-health-consequences-of-e-cigarettes.

[2] The Flavor and Extract Manufacturers Association of the United States Respiratory Health and Safety in the Flavor Manufacturing Workplace. https://www.femaflavor.org/sites/default/files/2018-06/FEMA%202012%20Respiratory%20Health%20and%20Safety%20in%20Workplace.pdf.

[3] Office for Health Improvement and Disparities Nicotine Vaping in England: 2022 Evidence Update. https://www.gov.uk/government/publications/nicotine-vaping-in-england-2022-evidence-update.

[4] Polosa R., O’Leary R., Tashkin D., Emma R., Caruso M. (2019). The effect of e-cigarette aerosol emissions on respiratory health: A narrative review. Expert Rev. Resp. Med..

[5] Soulet S., Sussman R.A. (2022). Critical review of the recent literature on organic byproducts in e-Cigarette aerosol emissions. Toxics.

[6] Hutzler C., Paschke M., Kruschinski S., Henkler F., Hahn J., Luch A. (2014). Chemical hazards present in liquids and vapors of electronic cigarettes. Arch. Toxicol..

[7] Girvalki C., Tzatzarakis M., Kyriakos C.N., Vardavas A.I., Stivaktakis P.D., Kavvalakis M., Tsatsakis A., Vardavas C. (2018). Composition and chemical health hazards of the most common electronic cigarette liquids in nine European countries. Inhal. Toxicol..

[8] Farsalinos K., Lagoumintzis G. (2019). Toxicity classification of e-cigarette flavouring compounds based on European Union regulation: Analysis of findings from a recent study. Harm Reduct. J..

[9] Bonner E., Chang Y., Christie E., Colvin V., Cunningham B., Elson D., Ghetu C., Huizenga J., Hutton S.J., Kolluri S.K. (2021). The chemistry and toxicology of vaping. Pharmacol. Ther..

[10] Omaiye E.E., Luo W., McWhirter K.J., Pankow J.F., Talbot P. (2020). Electronic cigarette refill fluids sold worldwide: Flavor chemical composition, toxicity and hazard analysis. Chemical Res. Toxicol..

[11] State and Community Tobacco Control Research. Standardized Tobacco Assessment for Retail Settings (STARS) Pocket Guide. http://countertobacco.org/wp-content/uploads/2017/02/5_STARS_Pocket_Guide.pdf.

[12] Buettner-Schmidt K., Miller D.R., Orr M., Balasubramanian N., Rykal K., Steward K.F., Swanson K., Berry M. (2021). Electronic cigarette refill liquids: Nicotine content, presence of child-resistant packaging, and in-shop compounding. J. Pediatr. Nurs..

[13] Melgar R., Kelly R.C. (1993). A novel GC/MS derivatization method for amphetamines. J. Anal. Toxicol..

[14] Young R.F., Coy D.L., Fedorak P.M. (2010). Evaluating MTBSTFA derivatization reagents for measuring naphthenic acids by gas chromatography-mass spectrometry. Anal. Methods.

[15] National Institute of Standards and Technology NIST Standard Reference Database 1A: NIST/EPA/NIH Mass Spectral Library (NIST 08) and NIST Mass Spectral Search Program (Version 2.0f). https://chemdata.nist.gov/mass-spc/ms-search/docs/Ver20Man.pdf.

[16] Schoene K., Bruckert H.J., Steinhanses J., König A. (1994). Two stage derivatization with N-(tert.-butyldimethylsilyl)- N-methyl-trifluoroacetamide (MTBSTFA) and N-methyl-bis-(trifluoroacetamide) (MBTFA) for the gas-chromatographic analysis of OH-, SH- and NH-compounds. J. Anal. Chem..

[17] Schummer C., Delhomme O., Appenzeller B., Wenning R., Millet M. (2009). Comparisons of MTBSTFA and BSTFA in derivatization reactions of polar compounds prior to GC/MS analysis. Talanta.

[18] Armbruster D.A., Pry T. (2008). Limit of blank, limit of detection and limit of quantitation. Clin. Biochem. Rev..

[19] Detection Limit/Quantitation Limit Summary Table. https://www.epa.gov/sites/default/files/2015-06/documents/mdlmql-toolbox-final-oct2010.pdf.

[20] Kim S., Chen J., Cheng T., Gindulyte A., He J., He S., Li Q., Shoemaker B.A., Thiessen P.A., Yu B. (2021). PubChem in 2021: New data content and improved web interfaces. Nucleic Acids Res..

[21] Wishart D.S., Tzur D., Knox C., Eisner R., Guo A.C., Young N., Cheng D., Jewell K., Arndt D., Sawhney S. (2007). HMDB: The Human Metabolome Database. Nucleic Acids Res..

[22] The Good Scents Company The Good Scents Company Information System: Providing Information for the Flavor, Fragrance, Food, and Cosmetic Industries. http://www.thegoodscentscompany.com/index.html.

[23] Flavor & Extract Manufacturers Association Flavor Ingredient Library. https://www.femaflavor.org/flavor-library.

[24] Human Metabolome Database: Showing Metabocard for Vanillin Acetate (HMDB0029663). https://hmdb.ca/metabolites/HMDB0029663.

[25] International Union of Pure and Applied Chemistry (IUPAC) Home Page. https://iupac.org/.

[26] CAS REGISTRY Chemical Abstract Services (CAS) Home Page. https://www.cas.org/cas-data/cas-registry.

[27] Krüsemann E.J.Z., Boesveldt S., de Graaf K., Talhout R. (2019). An e-liquid flavor wheel: A shared vocabulary based on systematically reviewing e-liquid flavor classifications in literature. Nicotine Tob. Res..

[28] Chong J., Xia J. (2018). MetaboAnalystR: An R package for flexible and reproducible analysis of metabolomics data. Bioinformatics..

[29] Scieszka D.P., Garland D., Hunter R., Herbert G., Lucas S., Jin Y., Gu H., Campen M.J., Cannon J.L. (2023). Multi-omic assessment shows dysregulation of pulmonary and systemic immunity to e-cigarette exposure. Respir. Res..

[30] Hsiao Y.C., Matulewicz R.S., Sherman S.E., Jaspers I., Weitzman M.L., Gordon T., Liu C.-W., Tang Y., Lu K., Bjurlin M.A. (2023). Untargeted Metabolomics to Characterize the Urinary Chemical Landscape of E-Cigarette Users. Chem. Res. Toxicol..

[31] Wishart D.S., Guo A., Oler E., Wang F., Anjum A., Peters H., Dizon R., Sayeeda Z., Tian S., Lee B.L. (2021). HMDB 5.0: The Human Metabolome Database for 2022. Nucleic Acids Res..

[32] Lu X., Chen L., Yuan J., Luo J., Luo J., Xie Z., Li D. (2022). User perceptions of different electronic cigarette flavors on social media: Observational study. J. Med. Internet Res..

[33] Truth Initiative. Menthol: Facts, Stats and Regulations. https://truthinitiative.org/research-resources/traditional-tobacco-products/menthol-facts-stats-and-regulations.

[34] Lui G., Jen Lin C., Yates C.R., Prasad G.L. (2021). Metabolomic analysis identified reduced levels of xenobiotics, oxidative stress, and improved vitamin metabolism in smokers switched to use electronic nicotine delivery system. Nicotine Tob. Res..

[35] Food and Drug Administration FDA Finalizes Enforcement Policy on Unauthorized Flavored Cartridge-Based E-cigarettes That Appeal to Children, Including Fruit and Mint. https://www.fda.gov/news-events/press-announcements/fda-finalizes-enforcement-policy-unauthorized-flavored-cartridge-based-e-cigarettes-appeal-children.

[36] Wickham R.J. (2020). The biological impact of menthol on tobacco dependence. Nicotine Tob. Res..

[37] Kopa P.N., Pawliczak R. (2020). Menthol additives to tobacco products. Reasons for withdrawing mentholated cigarettes in European Union on 20th May 2020 according to tobacco products directive (2014/40/EU). Toxicol. Mech. Methods.

[38] Chaiton M.O., Cunningham R., Hagen L., Dubray J., Borland T. (2022). Taking global leadership in banning menthol and other flavours in tobacco: Canada’s experience. Tob. Control.

[39] Food and Drug Administration Harmful and Potentially Harmful Constituents in Tobacco Products and Tobacco Smoke: Established List. https://www.fda.gov/tobacco-products/rules-regulations-and-guidance/harmful-and-potentially-harmful-constituents-tobacco-products-and-tobacco-smoke-established-list.

[40] Food and Drug Administration Harmful and Potentially Harmful Constituents (HPHCs). https://www.fda.gov/tobacco-products/products-ingredients-components/harmful-and-potentially-harmful-constituents-hphcs.

[41] National Institute for Public Health and the Environment RIVM Prepares a List of Banned Substances in Tobacco Products and E-Cigarettes. https://www.rivm.nl/en/news/rivm-prepares-recommended-list-of-banned-substances-in-tobacco-products-and-e-cigarettes.

[42] Moldoveanu S.C., Bussey R.O. (2023). Two techniques for the analysis of WS-3 with potential application to the analysis of other cooling agents. Curr. Issues Transp. Res..

[43] Leventhal A.M., Tackett A.P., Whitted L., Jordt S.E., Jabba S.V. (2022). Ice flavours and non-menthol synthetic cooling agents in e-cigarette products: A review. Tob. Control.

[44] Jabba S.V., Erythropel H.C., Garcia Torres D., Delgado L.A., Woodrow J.G., Anastas P.T., Zimmerman J.B., Jordt S.E. (2022). Synthetic cooling agents in us-marketed e-cigarette refill liquids and popular disposable e-cigarettes: Chemical analysis and risk assessment. Nicotine Tob. Res..

[45] Gschwend G., Jenkins C., Jones A., Kelso C., Morgan J. (2023). A wide range of flavoring-carrier fluid adducts form in e-cigarette liquids. Chem. Res. Toxicol..

[46] Erythropel H.C., Davis L.M., de Winter T.M., O’Malley S.S., Krishnan-Sarin S., Zimmerman J.B. (2019). Flavorant-solvent reaction products and menthol in JUUL e-cigarettes and aerosol. Am. J. Prev. Med..

[47] Narimani M., Adams J., da Silva G. (2022). Toxic chemical formation during vaping of ethyl ester flavor additives: A chemical kinetic modeling study. Chem. Res. Toxicol..

[48] Fiesel P.D., Kerwin R.A., Daniel Jones A., Last R.L. (2023). Trading acyls and swapping sugars: Metabolic innovations in *Solanum* trichomes. bioRxiv.

[49] Qi M., Ge X., Liang M., Fu R. (2004). Flash gas chromatography for analysis of volatile compounds from Houttuynia cordata Thunb. Anal. Chim. Acta.

[50] Chappell B. The FDA Orders Juul to Pull All of Its Vaping Products from the U.S. Market. https://www.npr.org/2022/06/23/1106846310/juul-fda-vaping-e-cigarettes.

