# Supplementary material for: Development of a Flavor Ingredient Wheel Linking E-Liquid Additives to the Labeled Flavor of Vaping Products
Source: Toxics. 2024 May 18;12(5):372. doi: 10.3390/toxics12050372 (PMC11125894; doi:10.3390/toxics12050372)
Supplement: Supplementary file 1 [file toxics-12-00372-s001.zip › toxics-2761318-Supplementary file.pdf]

toxics

Article

Development of a Flavor Ingredient Wheel Linking E-Liquid Additives to the Labeled Flavor of Vaping Products

Kelly Buettner-Schmidt [1,*], Katherine Steward [2], Maciej L. Goniewicz [3], Kolby Schaeffer Fraase [1], Megan Orr [1] and Donald R. Miller [1]

[1] School of Nursing, North Dakota State University, Fargo, ND 58108, USA; kolby.schaeffer.1@ndsu.edu (K.S.F.); megan.orr@ndsu.edu (M.O.); donald.r.miller@ndsu.edu (D.R.M.)
[2] Department of Chemistry and Biochemistry, Montana State University, Bozeman, MT 59717, USA; kfsteward@gmail.com
[3] Department of Health Behavior, Roswell Park Comprehensive Cancer Center, Buffalo, NY 14263, USA; maciej.goniewicz@roswellpark.org
* Correspondence: kelly.buettnerschmi@ndsu.edu; Tel.: +1-701-231-8232

Citation: Buettner-Schmidt, K.; Steward, K.; Goniewicz, M.L.; Schaeffer Fraase, K.; Orr, M.; Miller, D.R. Development of a Flavor Ingredient Wheel Linking E-Liquid Additives to the Labeled Flavor of Vaping Products. *Toxics* **2024**, *12*, 372. https://doi.org/10.3390/toxics12050372

Academic Editors: Teresa D. Tetley and Edmund Maser

Received: 22 November 2023
Revised: 11 May 2024
Accepted: 13 May 2024
Published: 18 May 2024

Copyright: © 2024 by the authors. Licensee MDPI, Basel, Switzerland. This article is an open access article distributed under the terms and conditions of the Creative Commons Attribution (CC BY) license (https://creativecommons.org/licenses/by/4.0/).

Table S1. Integrated Peak Area Abundances for LOQ Estimation.

Chemical Name/Ingredient	3XLOD Estimate (Abundance)
3-Hexen-1-ol, (Z)-	Resp. >3000
1,3-Dioxolane, 4-methyl-2-phenyl- Results	Resp. >1000
a-Nicotine Results	Resp. >5000
2-Cyclohexen-1-one, 2-methyl-5-(1-methylethenyl)-, (R)- (Carvone) (anise) Results	Resp. >1000
1,3-Benzodioxole, 5-(4-methyl-1,3-dioxolan-2-yl)- (Heliotropine propylene glycol acetal) (apple) Results	Resp. >1000
Hexanoic acid, butyl ester (Butyl caproate- apricot) Results	Resp. >3000
Butanoic acid, 2-methyl-, ethyl ester (apple, apricot, orange, grapefruit) Results	Resp. >1000
Butanoic acid, 2-methyl- (Active Valeric Acid) (fruity) Results	Resp. >2000
Butanoic acid, 3-methyl-, ethyl ester (apple) Results	Resp. >2000
1-Butanol, 3-methyl-, acetate (Banana/ pear) Results	Resp. >1000
4H-Pyran-4-one, 2-ethyl-3-hydroxy- Ethyl maltol (caramelized sugar and cooked fruit) Results	Resp. >2000
Benzene, 1-methyl-2-(1-methylethyl)- (o-Cymene) (citrusorange) Results	Resp. >1000
2H-1-Benzopyran-2-one, 6-methyl- (coconut) Results	Resp. >2000
1,3-Dioxolane-2-propanoic acid, 2,4-dimethyl-, ethyl ester Results	Resp. >2000
Veratraldehyde propylene glycol acetal (ethyl vanillin) Results	Resp. >1000
Ethyl Vanillin Results	Resp. >2000
Benzyl Alcohol (flavor enhancer) Results	Resp. >500
1,3-Dioxan-5-ol, 2-phenyl- (Benzaldehyde glyceryl acetal derivitive) food flavor Results	Resp. >2000
Acetic acid, pentyl ester (pear and apple scent) Results	Resp. >1000
Acetic acid, hexyl ester (fruit essence) Results	Resp. >10000
Pentanoic acid, 4-oxo-, 1-methylethyl ester (iso-Propyl levulinate) fruity Results	Resp. >10000
Benzoic acid, 2-amino-, methyl ester (grape and many others) Results	Resp. >2000
2-Propenoic acid, 3-phenyl-, methyl ester (Cinnamic acid, methyl ester, (E)-) (guava, feijoa, strawberry) Results	Resp. >30000
Butanoic acid, 3-methyl-, propyl ester (banana, jackfruit and gruyere) Results	Resp. >2000
(S)-2-Hydroxypropanoic acid (Lactic acid) Results	Resp. >30000
1,4-Dioxane-2,5-dione, 3,6-dimethyl- (lactide) Results	Resp. >50000
Pentanoic acid, 4-oxo-, butyl ester (levulinic acid) Results	Resp. >50000
Limonene (lemon) Results	Resp. >1000
5-Thiazoleethanol, 4-methyl- (meaty flavor) Results	Resp. >2000
Cyclohexanol, 1-methyl-4-(1-methylethyl)- (menthol) Results	Resp. >1000
N-Acetyl-4-(2',3'-dihydroxypropoxy)phenylacetamide Results	Resp. >100000
N-Ethyl-4-hydroxypiperidine Results	Resp. >100000
Neonicotine Results	Resp. >2000
(1's,2's)-Nicotine-N'-oxide Results	Resp. >500
Nicotine Results	Resp. >500
Non-7-enoic acid, dimethylamide Results	Resp. >15000
Triacetin (papaya) Results	Resp. >2000

(2(3H)-Furanone, 5-heptyldihydro-) Peach lactone Results	Resp. >500
2(3H)-Furanone, 5-hexyldihydro- (peach) Results	Resp. >1000
Benzyl Benzoate (perfume fixative as well as: scabicide, an acaricide and a plant metabolite) Results	Resp. >1000
1,2,3-Propanetriol, monoacetate/ Acetin (perfume fixative) Results	Resp. >5000
1R-a-Pinene (pine or rosemary) Results	Resp. >1000
Salicylic acid Results	Resp. >1000
2H-1-Benzopyran-2-one, 3,4-dihydro- (sour cherry, terragon) Results	Resp. >1000
Oxiranecarboxylic acid, 3-methyl-3-phenyl-, ethyl ester, cis- (Hydrocinnamic acid) (strawberry) Results	Resp. >500
1-Deoxy-d-arabitol (sugar alcohol) Results	Resp. >10000
2H-Pyran-2-one, 6-heptyltetrahydro- (tropical) Results	Resp. >1000
Pentanoic acid, 2-acetyl-4-methyl-, ethyl ester (valeric acid) Results	Resp. >10000
Vanillin, acetate Results	Resp. >10000
3-Buten-2-one, 4-(2,6,6-trimethyl-1-cyclohexen-1-yl)-, (E)- (ionone) (violets) Results	Resp. >1500

Note. LOQ estimation based on manual LOD peak area determinations. Any response greater than the listed response would theoretically be quantifiable and was thus counted as a "positive detection" for that ingredient.

Figure S1. Average Chemical Classification Breakdown of Ingredients by Labeled Flavor Category.

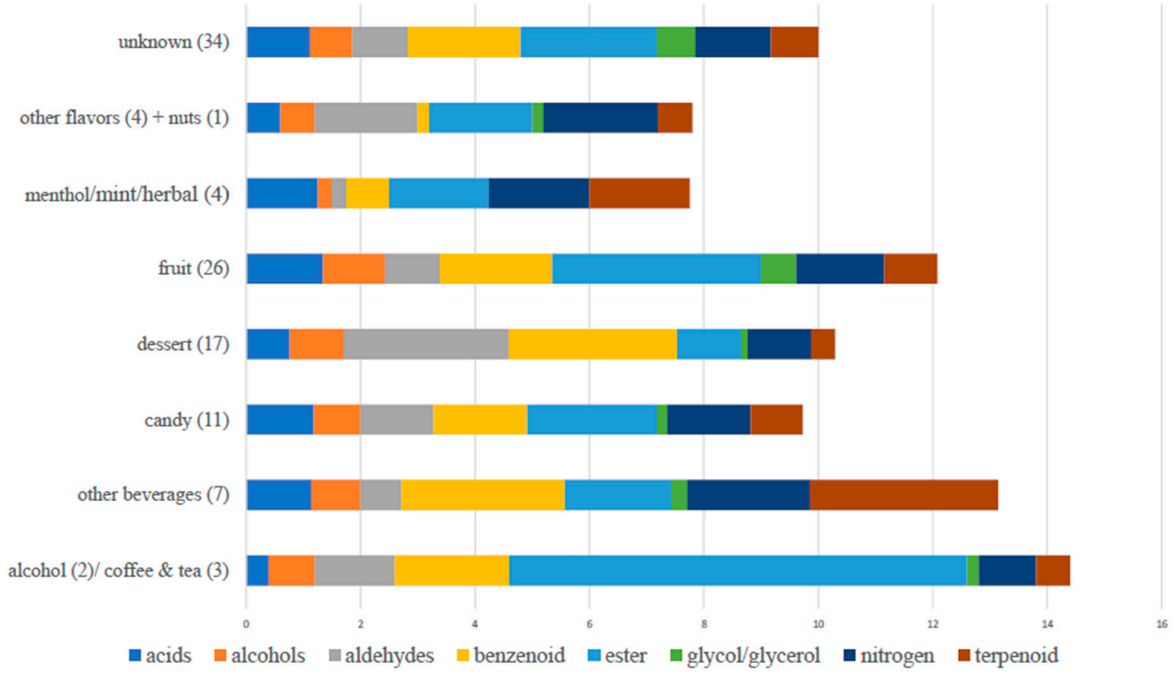

Note. Using the root chemical class for each ingredient and the generalized flavor category of the samples, the mean number of ingredients for each flavor are represented as a stacked bar plot. For each flavor category, the mean number of ingredients belonging to each root chemical class was determined and plotted to represent the average makeup of each flavor.

Figure S2. Ingredients by Flavor Classification.

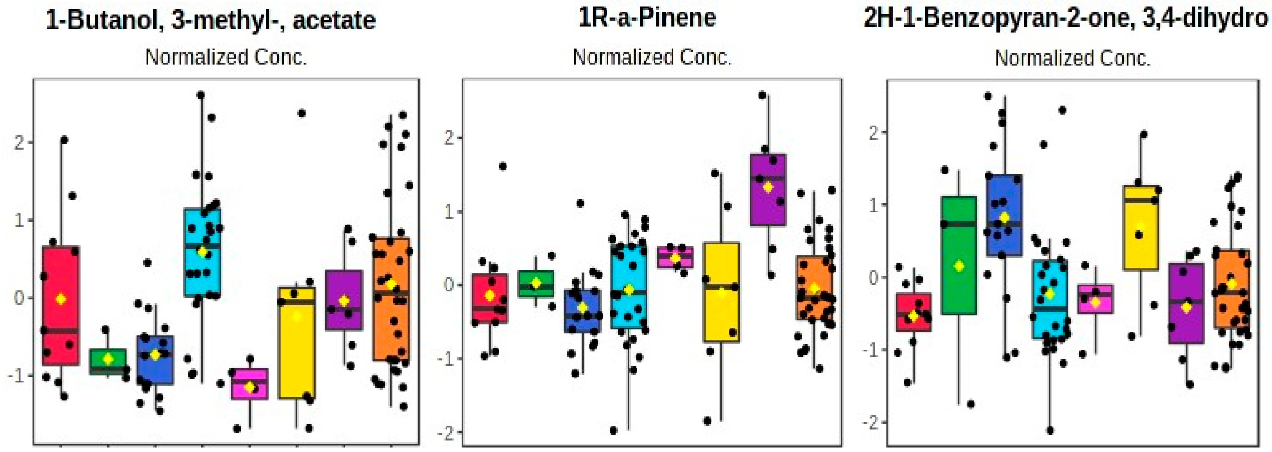

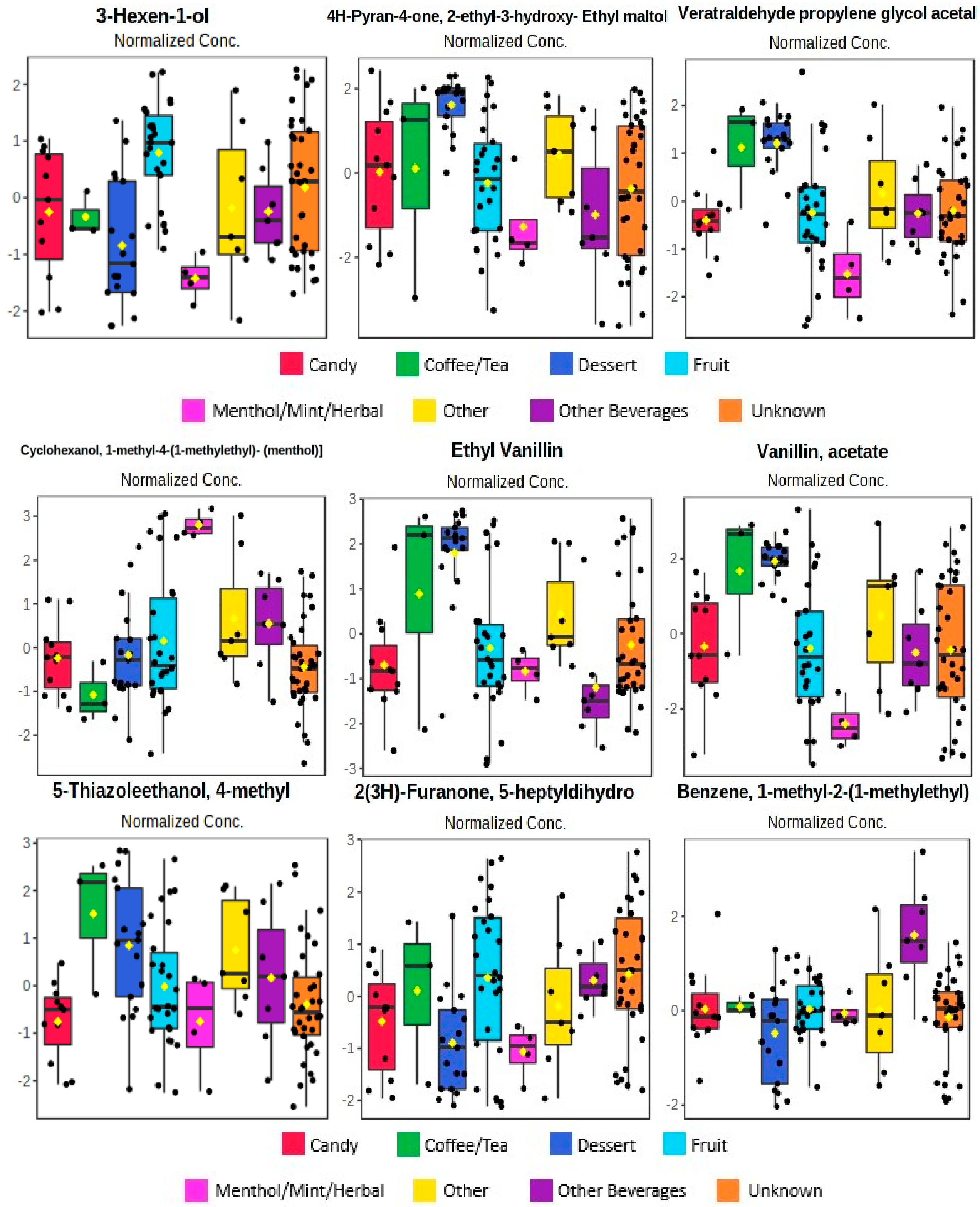
3-Hexen-1-ol
Normalized Conc.
4H-Pyran-4-one, 2-ethyl-3-hydroxy- Ethyl maltol
Normalized Conc.
Veratraldehyde propylene glycol acetal
Normalized Conc.
Candy
Coffee/Tea
Dessert
Fruit
Menthol/Mint/Herbal
Other
Other Beverages
Unknown
Cyclohexanol, 1-methyl-4-(1-methylethyl)- (menthol)]
Normalized Conc.
Ethyl Vanillin
Normalized Conc.
Vanillin, acetate
Normalized Conc.
5-Thiazoleethanol, 4-methyl
Normalized Conc.
2(3H)-Furanone, 5-heptyldihydro
Normalized Conc.
Benzene, 1-methyl-2-(1-methylethyl)
Normalized Conc.
Candy
Coffee/Tea
Dessert
Fruit
Menthol/Mint/Herbal
Other
Other Beverages
Unknown

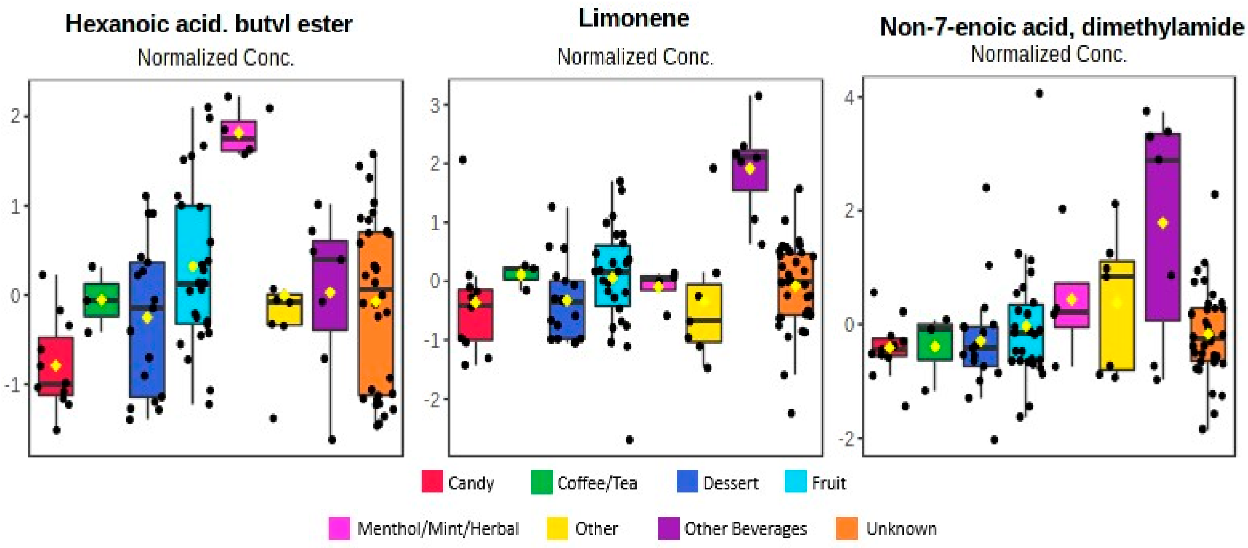

Note. The 15 significantly different ingredients by analysis of variance (ANOVA) test represented as box plots, with their common name displayed, groups: candy = red, coffee/tea = green, dessert = blue, fruit = aqua, menthol/mint/herbal = purple, other = yellow, other beverages = purple, and unknown = orange. Conc. = concentration.

Table S2. Ingredients With Significant Mean Abundance Differences Among Flavor Categories.

Ingredient	*p* value
Ethyl vanillin	2.48×10^{-6}
Limonene	3.40×10^{-6}
Vanillin, acetate	4.35×10^{-6}
Veratraldehyde propylene glycol acetal	4.50×10^{-6}
Cyclohexanol, 1-methyl-4-(1-methylethyl)- (menthol)]	8.39×10^{-5}
3-hexen-1-ol	0.00011589
1R-a-pinene	0.00022557
Hexanoic acid, butyl ester	0.00023269
4H-pyran-4-one, 2-ethyl-3-hydroxy- ethyl maltol	0.00037063
1-butanol, 3-methyl-, acetate	0.00047774
2H-1-benzopyran-2-one, 3,4-dihydro	0.00063365
Benzene, 1-methyl-2-(1-methylethyl)	0.00064589
Non-7-enoic acid, dimethylamide	0.00098517
5-thiazoleethanol, 4-methyl	0.0026388
2(3H)-furanone, 5-heptyldihydro	0.0086826

Note. *p* values determined using one-way analysis of variance.

Figure S3. Heatmap of Flavor Category.

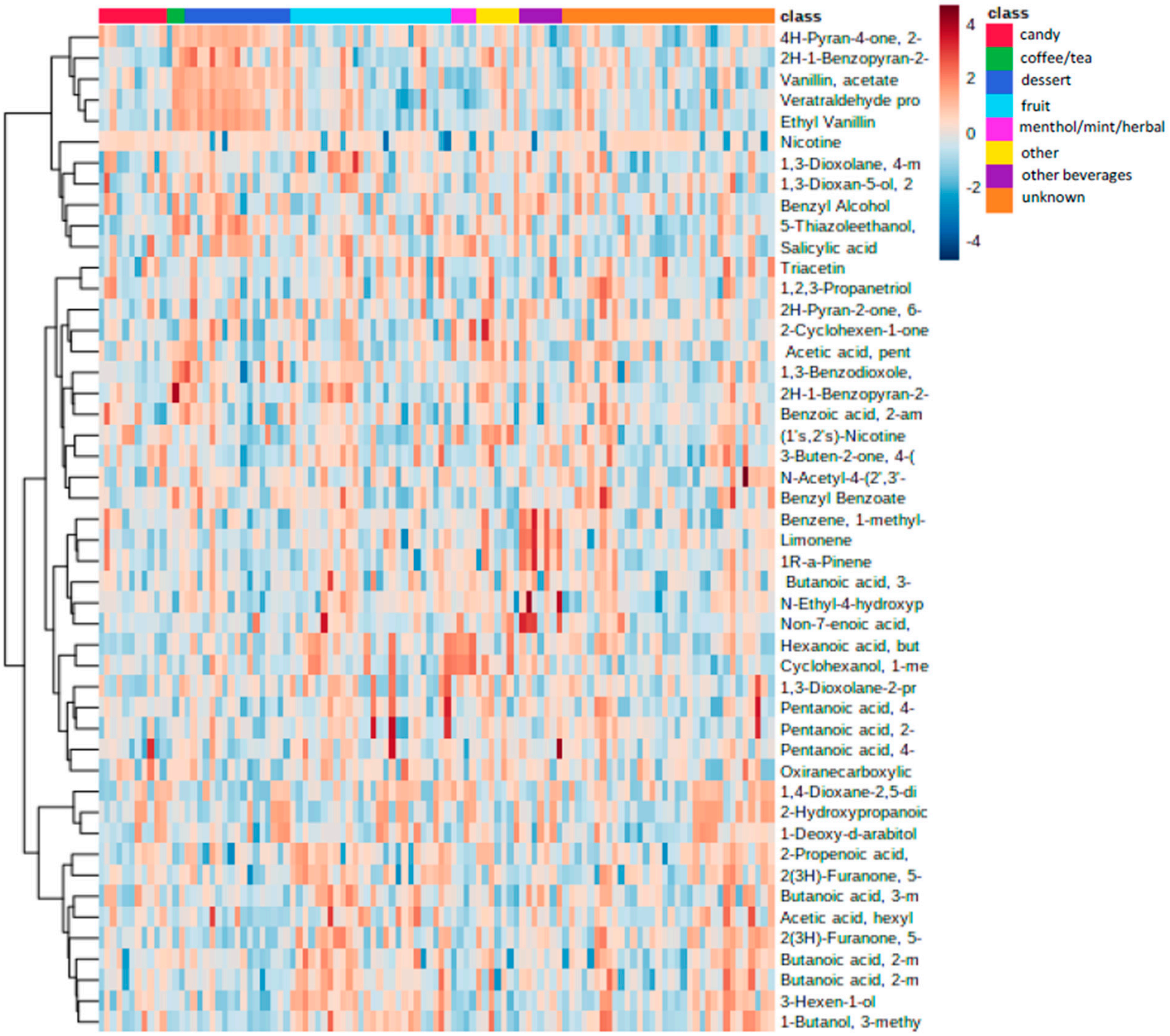

Note. Heatmap showing the relative abundances using the red/blue color scale of ingredients detected in e-liquids. All ingredients and all 109 samples are represented, with grouping based on flavor description (candy = red, coffee/tea = green, dessert = blue, fruit = teal, menthol/mint/herbal = pink, other = yellow, other beverages = purple, and unknown =orange).

Table S3. Ingredients Significantly Different Between Groups Based on Cooling Claim.

Ingredient	FC	*p*-value
cyclohexanol, 1-methyl-4-(1-methylethyl)- (menthol)]	−41.848	7×10^{-10}
hexanoic acid, butyl ester	−8.78735	7.10×10^{-6}
vanillin, acetate	26.187	4.80×10^{-4}
non-7-enoic acid, dimethylamide	−31.2061	3.00×10^{-3}
2H-1-benzopyran-2-one, 3,4-dihydro	13.64	6.90×10^{-3}
4H-pyran-4-one, 2-ethyl-3-hydroxy- ethyl maltol	10.142	1.00×10^{-2}
veratraldehyde propylene glycol acetal	7.7431	1.40×10^{-2}
benzoic acid, 2-amino-, methyl ester	20.09	3.00×10^{-2}
butanoic acid, 2-methyl-, ethyl ester	3.6975	4.80×10^{-2}

Note. The log fold change and p-value for ingredients with significant differences ($p < 0.05$) in mean relative abundance between samples with a cooling claim and those without a cooling claim.

Figure S4. Partial Least Squares Discriminant Analysis and Variable of Importance in Projection Ingredients Based on Cooling Claim on Packaging.

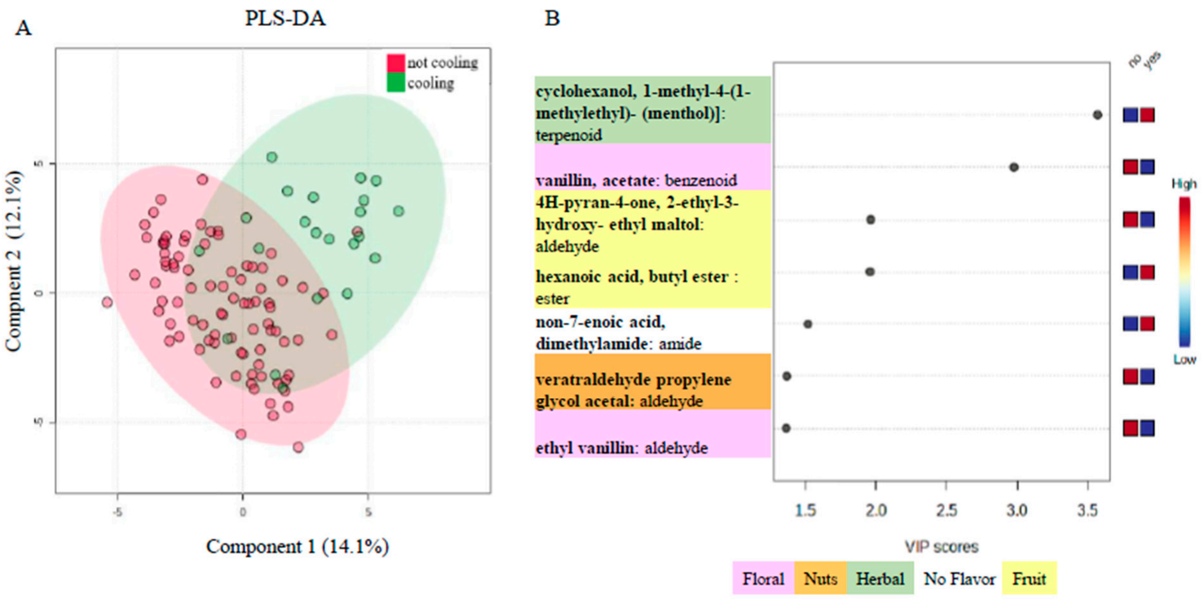

Note. (**A**) A partial least squares discriminant analysis (PLS-DA) demonstrating the dimensional reduction of the patterns in the ingredient data based on cooling description. Samples that claim no cooling (red) cluster closer together with less variability than samples that claim a cooling effect (green). (**B**) Variable importance in projection (VIP) scores of ingredients in e-liquid that contribute to the variation between liquid type as displayed in the PLS-DA plot. Relative abundances of ingredients are displayed in the blue/red gradient to the right of the chart. Ingredient and root chemical class are displayed, with flavor profile represented by color as shown below the plot.

Figure S5. Bar Plot of Distribution of Detected E-Liquid Ingredients.

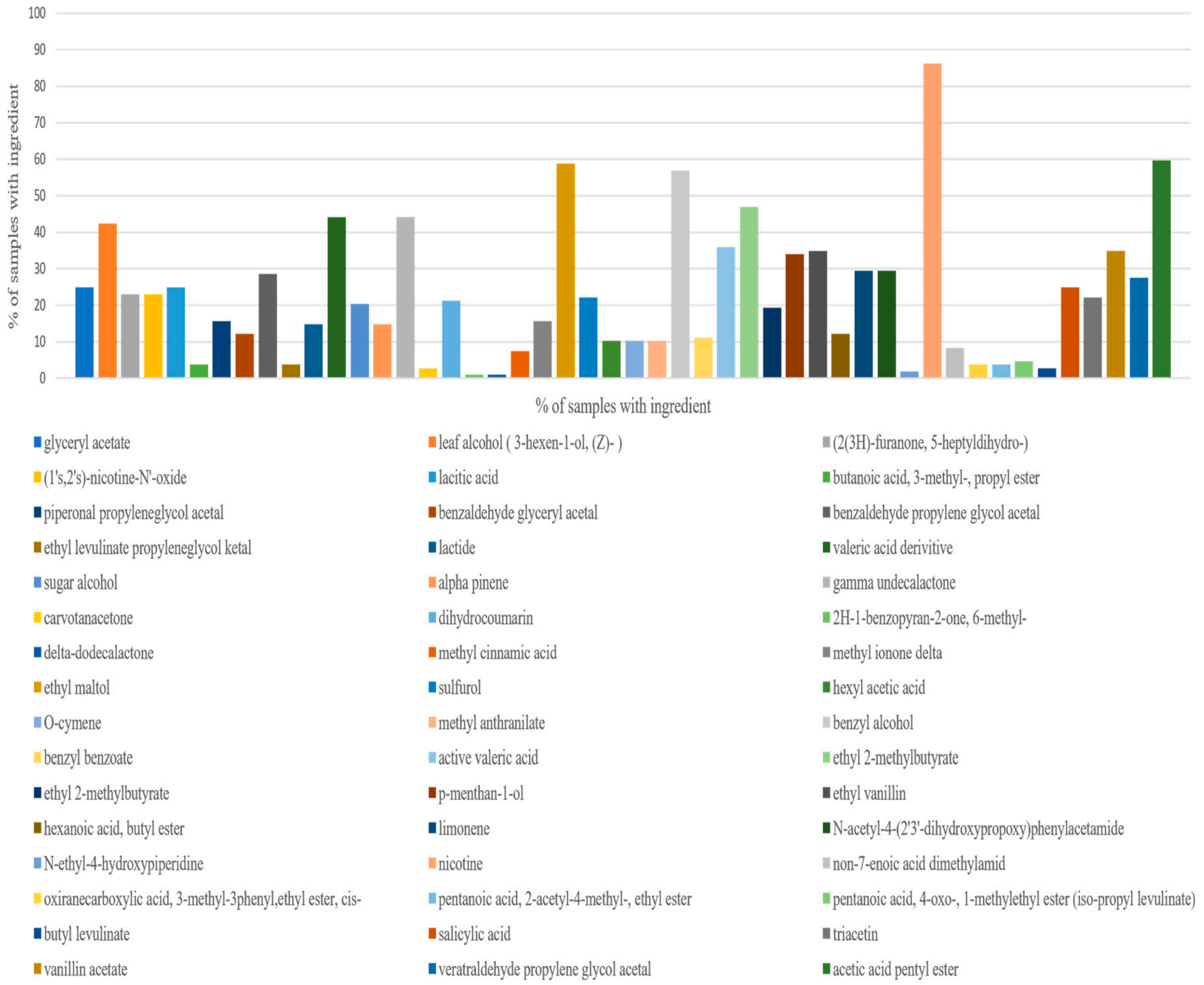

Note. Bar plot showing the percentage of analyzed products (out of 109 samples) that were positive for individual ingredients.

Table S4. Detected Acid Ingredients.

	Samples, n (%)	Nicotine Type (based on product label)			Proportion of + salt samples, %	Proportion of + free-base samples, %	Proportion of + 0 nicotine samples, %
		Salt, n	Free-base, n	0 nicotine, n			
**levulinic acid**	**3 (2.8)**	**3**	**0**	0	100	0	0
**lactic acid**	27 (24.8)	26	1	0	96.3	3.7	0
**salicylic acid**	27 (24.8)	11	10	6	40.7	37	22.2
**benzoic acid**	11 (10.1)	5	4	2	45.5	36.4	18.2
**levulinic + salicylic**	1 (0.9)						
**lactic + salicylic**	8 (7.3)						
**lactic + benzoic**	1 (0.9)						
**salicylic +benzoic**	2 (1.8)						

**lactic + salicylic + benzoic**	2 (1.8)	

Note. Each identified acid ingredient and the number of samples that had a positive detection were determined, and the proportions of each sample relative to a total number of samples and different sample groups.

Table S5. Comparison of Primary Flavor Categories.

	**Krüsemann et al.'s Flavor Wheel**	**Proposed in the Ingredient Wheel**
1	Tobacco	Not included
2	Menthol/Mint	Menthol/mint/herbal
3	Nuts	Not included
4	Spices	Not included
5	Coffee/Tea	Not included
6	Alcohol	Not included
7	Other Beverages	Not included
8	Fruit	Fruit
9	Dessert	Dessert
10	Candy	Candy
11	Other Sweets	Floral
12	Other Flavors	Other
13	Unflavored	Not included
	Not included	Nicotine

Note. Krüsemann EJZ, Boesveldt S, de Graaf K, Talhout R. An e-liquid flavor wheel: a shared vocabulary based on systematically reviewing e-liquid flavor classifications in literature. *Nicotine & Tobacco Research*. 2019;21(10):1310-1319. doi: 10.1093/ntr/nty101 *Characterizing Product Flavors Using the Flavor Ingredient Wheel.*

Table S6. Characterizing Product Flavors Using the Flavor Ingredient Wheel.

**Fruit/Berry - Not Cooling**	
3-hexen-1-ol: alcohol	
Benzoic acid, 2-amino-, methyl ester: ester	
2(3H)-furanone, 5-heptyldihydro: benzenoid	
**Dessert 1 - Cooling**	**Dessert 2 - Not Cooling**
1,3-dioxolane, 4-methyl-2-phenyl: acid	1,3-dioxolane, 4-methyl-2-phenyl: acid
Hexanoic acid, butyl ester: ester	1,3-benzodioxole, 5-(4-methyl-1,3-dioxolan-2-yl): glycol
4H-pyran-4-one, 2-ethyl-3-hydroxy- Ethyl maltol: aldehyde	4H-pyran-4-one,2-ethyl-3-hydroxy-Ethyl maltol: aldehyde
Veratraldehyde propylene glycol acetal: aldehyde	Veratraldehyde propylene glycol acetal: aldehyde
Ethyl vanillin: aldehyde	Ethyl vanillin: aldehyde
Benzyl alcohol: benzenoid	Benzyl alcohol: benzenoid
1,3-dioxan-5-ol, 2-phenyl- (benzaldehyde glyceryl acetal derivative): aldehyde	Benzoic acid, 2-amino-, methyl ester: ester
2-hydroxypropanoic acid: acid	Salicylic acid: acid
Cyclohexanol,1-methyl-4-(1-methylethyl)- (menthol)]: terpenoid	Vanillin, acetate: benzenoid
Triacetin: glycerol	

1,2,3-propanetriol,monoacetate: glycol/glycerol/sugar	
Salicylic acid: acid	
1-deoxy-d-arabitol (sugaralcohol): glycol/glycerol/sugar	
Vanillin, acetate: benzenoid	
**Fruit - Other 1**	**Fruit - Other 2**
1,3-dioxolane, 4-methyl-2-phenyl: acid	3-hexen-1-ol: alcohol
1,3-benzodioxole, 5-(4-methyl-1,3-dioxolan-2-yl): glycol	Butanoic acid, 2-methyl-, ethyl ester: ester
Butanoic acid, 2-methyl-, ethyl ester: ester	Butanoic acid, 2-methyl-: ester
Butanoic acid, 2-methyl-: ester	Butanoic acid, 3-methyl-, ethyl ester: ester
1-butanol, 3-methyl-, acetate: ester	1-butanol, 3-methyl-, acetate: ester
4H-pyran-4-one, 2-ethyl-3-hydroxy- Ethyl maltol: aldehyde	4H-pyran-4-one,2-ethyl-3-hydroxy-Ethyl maltol: aldehyde
1,3-dioxan-5-ol, 2-phenyl- (benzaldehyde glyceryl acetal derivative): aldehyde	Benzyl Alcohol: benzenoid
Benzoic acid, 2-amino-, methyl ester: ester	2-propenoic acid, 3-phenyl-, methyl ester (cinnamic acid): ester
2-hydroxypropanoic acid: acid	2-hydroxypropanoic acid: acid
Triacetin: glycerol	1,4-dioxane-2,5-dione, 3,6-dimethyl: acid
1,2,3-propanetriol, monoacetate: glycol/glycerol/sugar	1-deoxy-d-arabitol (sugar alcohol): glycol/glycerol/sugar

Legend:

Floral	Nuts	Herbal	No Flavor	Fruit

Disclaimer/Publisher's Note: The statements, opinions and data contained in all publications are solely those of the individual author(s) and contributor(s) and not of MDPI and/or the editor(s). MDPI and/or the editor(s) disclaim responsibility for any injury to people or property resulting from any ideas, methods, instructions or products referred to in the content.
